# Recent Advances in Nanosystems and Strategies for Vaginal Delivery of Antimicrobials

**DOI:** 10.3390/nano11020311

**Published:** 2021-01-26

**Authors:** Giulia Chindamo, Simona Sapino, Elena Peira, Daniela Chirio, Marina Gallarate

**Affiliations:** Department of Drug Science and Technology, University of Turin, 10125 Turin, Italy; giulia.chindamo@unito.it (G.C.); elena.peira@unito.it (E.P.); daniela.chirio@unito.it (D.C.); marina.gallarate@unito.it (M.G.)

**Keywords:** vaginal infections, antimicrobials, drug delivery systems, nanocarriers, local therapies

## Abstract

Vaginal infections such as bacterial vaginosis (BV), chlamydia, gonorrhea, genital herpes, candidiasis, and trichomoniasis affect millions of women each year. They are caused by an overgrowth of microorganisms, generally sexually transmitted, which in turn can be favored by alterations in the vaginal flora. Conventional treatments of these infections consist in systemic or local antimicrobial therapies. However, in the attempt to reduce adverse effects and to contrast microbial resistance and infection recurrences, many efforts have been devoted to the development of vaginal systems for the local delivery of antimicrobials. Several topical dosage forms such as aerosols, lotions, suppositories, tablets, gels, and creams have been proposed, although they are sometimes ineffective due to their poor penetration and rapid removal from the vaginal canal. For these reasons, the development of innovative drug delivery systems, able to remain in situ and release active agents for a prolonged period, is becoming more and more important. Among all, nanosystems such as liposomes, nanoparticles (NPs), and micelles with tunable surface properties, but also thermogelling nanocomposites, could be exploited to improve local drug delivery, biodistribution, retention, and uptake in vulvovaginal tissues. The aim of this review is to provide a survey of the variety of nanoplatforms developed for the vaginal delivery of antimicrobial agents. A concise summary of the most common vaginal infections and of the conventional therapies is also provided.

## 1. Introduction

The vagina is a muscle-membranous canal that extends from the vulva to the cervix. The wall of the vagina includes different layers: an inner mucosal tissue, consisting of a stratified squamous epithelium, a middle smooth muscle layer made of longitudinal and circular fibers, an external coat of connective tissue, and the peritoneum [1]. The vagina is involved in several functions such as sexual functions, but also in active immunologic and anatomically mediated processes essential for maintaining a microenvironment ideal for “normal” bacteria [2]. In particular, many studies have demonstrated that several immune-related cells and receptors are present in the vagina, helping the microbial environment. There are five major types of vaginal microbiota, known as community state types (CSTs). In four of these (CST-I, -II, -III, -V), *Lactobacillus* spp. is the predominant species; CST-IV, instead, is composed of a mixture of several facultative anaerobes, including *Gardnerella*, *Atopobium*, and *Prevotella*. The frequency of these CSTs is different according to the ethnic backgrounds. Alterations in normal composition and functions of vaginal flora are involved in the development of several vaginal diseases such as bacterial, fungal, and viral vaginosis and can also increase the risk of acquiring sexually transmitted infections such as HIV, as demonstrated by some studies [3].

Female reproductive tract-related diseases to (FRT) affect more than 10 million people each year. Their symptoms often include itching, burning, pain, and dyspareunia, and are responsible for significant distress for patients [4].

Standard treatment options are numerous and mainly consist in antimicrobial drugs, systemically administered, although topical treatments can also be prescribed. Some clinical trials comparing oral with topical route demonstrated that women experienced similar cure rate, but those using the intravaginal antimicrobial agents resulted in being more satisfied with the treatment [5,6].

In addition, several oral antimicrobial therapies are responsible for side effects, including gastrointestinal disorders and candida infection. Moreover, low adherence to oral therapy may determine treatment failure, which in turn can increase the risk of recurrence or drug resistance. On the contrary, intravaginal treatments offer several advantages, including the capability of by-passing first metabolism, the ease of administration, and the reduction of side effects. 

Many researchers have shown that the vagina is an underutilized route for drug administration, and therefore remarkable efforts have recently been made to optimize this promising route of administration [7,8] for both local and systemic actions. In fact, systemic vaginal absorption of a variety of therapeutic molecules (proteins, small interfering RNAs, peptides, vaccines, oligonucleotides, antigens, and hormones) have been explored in recent years [9].

To date, a large number of topical dosage forms such as lotions, aerosols, gels, creams, suppositories, ovules, and tablets have been proposed for vaginal drug delivery but their effectiveness is highly limited by poor permeability across the vaginal wall and rapid removal from the vaginal canal, further influenced by the presence of hormones [10]. Accordingly, multiple and frequent local administrations are required to compensate for the rapid removal of drug carrier, leading to an increment of drug side effects and a reduction of patient compliance.

For these reasons, the vaginal delivery of drugs remains a challenge, and the discovery of novel effective local treatments able to remain in situ and release active agents for a prolonged period of time is gradually gaining interest [11].

In the first part of this review, we propose a focus on the main vaginal infections and their conventional treatments, while the second part focuses on the most recent innovative strategies, particularly the nanoplatforms, developed to optimize the topical vaginal delivery of antimicrobials.

## 2. Vaginal Infections and Conventional Treatments 

The vagina can be affected by several inflammations or infections caused mainly by bacteria but also by yeast, viruses, and parasites. Moreover, there are several associated risk factors such as sociodemographic characteristics, routine hygienic practices, hormonal imbalance, and lifestyle-related behaviors. A summary of the most commonly encountered vaginal infections, pathogens, and conventional treatments is presented in Table 1.

### 2.1. Bacterial Infections

Bacterial vaginosis (BV) is a common cause of medical problems whose exact etiology remains uncertain, although an interaction between microbial ecosystem and human host seems to occur; in particular, *Gardnerella vaginalis* replaces the resident *Lactobacillus* species. Risk factors are represented by vaginal douching; African American race; and, above all, multiple sexual partners. On the contrary, the use of hormonal contraceptive or condom could reduce the incidence of BV. Generally, BV treatment involves the use of antibacterial drugs, [12] especially metronidazole, tinidazole, secnidazole, and clindamycin. Furthermore, some alternative approaches such as the employment of probiotics/prebiotics, the local acidification, or the use of essential oils are under study [13].

Chlamydia is a sexually transmitted bacterial infection due to *Chlamydia trachomatis*, a Gram-negative obligate pathogen. In about 80% of affected women, chlamydia is asymptomatic, and in the other cases, vaginal discharge, mucopurulent endocervical discharge, and easily induced endocervical bleeding are the most common symptoms. If untreated, the infection may last for 4 years. Furthermore, chlamydia infections can lead to infertility, chronic pelvic pain, and ectopic pregnancy, and can also increase the risk of developing cervical cancer [14]. The first-choice treatment is based on the oral administration of azithromycin (1 g oral single dose) or doxycycline (100 mg orally twice a day for 7 days). However, azithromycin is not available in some settings, and thus erythromycin, ofloxacin, or levofloxacin can be administered as alternatives. Erythromycin exhibits noteworthy side effects that may reduce compliance, and levofloxacin and ofloxacin are high-cost drugs [15].

Gonorrhea is a sexually transmitted disease due to *Neisseria gonorrhoeae*, which is a Gram-negative diplococcus. It is often asymptomatic in females, but dysuria, vaginal discharge, abnormal uterine bleeding, dyspareunia, lower abdominal, and rectal pain are usually detected as symptoms. Furthermore, without a proper treatment, pelvic inflammatory disease, chronic pain, and facilitation of HIV transmission can be observed [16]. First-line treatment consists in the use of antibiotics (cefixime or ceftriaxone) administered orally or intramuscularly. In cases of drug resistance, a dual antibiotic treatment could be a possible option, even though both approaches often fail. After all, *Neisseria gonorrhoeae* antimicrobial resistance is an increasing issue, and for this reason, new antibiotics are being developed, e.g., macrolide drugs, tetracycline derivatives, and broad-spectrum fluoroquinolones [17,18].

Syphilis is caused by the bacterium *Treponema pallidum* subspecies pallidum and, like gonorrhea and chlamydia, it is a sexually transmitted infection; it can also be passed to the fetus during pregnancy or during vaginal birth. Syphilis is divided into three stages, with the first being characterized by a painless ulcer in which the infectious bacteria are located. The sore can last for several weeks, during which the infection can spread in cases of contact. If untreated, the primary syphilis can evolve to secondary syphilis, characterized by a rash diffused on several parts of the body that is sometimes accompanied by fever, fatigue, weight loss, enlarged lymph nodes, etc. Infected and untreated people can develop the third stage of syphilis, characterized by severe damages to vital organs and consequently problems such as dementia, paralysis, and loss of sight.

Furthermore, in a pregnant woman, untreated syphilis represents a potential risk to the fetus, and it is also well recognized as interacting synergistically with HIV [19].

The treatment of syphilis depends on the stage of infection, with *Treponema pallidum* being particularly susceptible to penicillin. Indeed, the standard therapy consists in benzathine penicillin G (2.4 million U) intramuscularly administered in three weekly doses; intramuscular ceftriaxone or oral tetracyclines are also considered, but there is less evidence of their efficacy. If all other options are not feasible, azithromycin in single 2 g oral dose is suggested [20].

Overall, chlamydia, gonorrhea, and syphilis are the most world spread sexually transmitted infections—all are caused by bacteria and are generally curable with antibiotics, but unfortunately the antibiotic resistance of these pathogens is rapidly growing especially in the case of gonorrhea. In this context, prompt treatment, prevention campaigns, and novel treatment options are becoming crucial. 

### 2.2. Viral Infections

Herpes genitalis is a sexually transmitted disease caused by the *Herpes simplex* virus (HSV), which can be subdivided in two types: HSV-1 (whose diffusion is growing) and HSV-2 (the most common form). HSV-1 infections mostly occur during childhood, while HSV-2 infections generally rise after puberty. Furthermore, herpes genitalis can manifest as primary or recurrent infection. Burning pain, fever, cervicitis, and lymphadenopathy are among the most common symptoms. Factors such as fever, UV light, stress or trauma, and immunogenic predisposition can act as triggers. Nevertheless, both symptoms and clinical recurrence are less severe in comparison with primary infection. First-line treatments are antivirals like acyclovir, valacyclovir, and famciclovir.

Systemically administered foscarnet and cidofovir (off-label) have been considered as alternative treatments to overcome resistance episodes. Unfortunately, they exhibit several adverse reactions such as urogenital ulcers, renal dysfunction, and nephrotoxicity. However, topical application of cidofovir gel or 1% foscarnet cream represents an attractive tool for the treatment of herpes genitalis. Currently, a new class of drugs called “helicase blockers” is under study, showing good results in preliminary tests [21,22].

*Human papilloma virus* (HPV) is probably the most common sexually transmitted infection in the world, improving the risk of cervical cancer and genital warts when the infection persists. HPV could infect squamous cells such as vaginal and anal epithelium, but also skin, oral, and nasal epithelium. The treatment for HPV infections is limited and influenced by viral latency and recurrent reinfections that reduce in a significant way the success of the treatment. In presence of a benign HPV lesion (genital warts), tissue destruction could be reached through thermal, electric, or chemical means. Four to six weeks are required before expecting benefit. Several strategies have been studied for the treatment of HPV infections. Among all, trichloroacetic acid, podophyllin derivatives, and imiquimod are the most used agents for chemical therapy, but other approaches, e.g., cryotherapy, laser, electrocautery, and surgical excision, are also diffused [23]. However, all above-mentioned treatments exhibit drawbacks such as short-term pain from healing to hypopigmentation and scarring; rarely, hyperesthesia, chronic pain, and dyspareunia are observed. 

AIDS (acquired immune deficiency syndrome) is probably one of the most dangerous human diseases. It is caused by the *Human immunodeficiency virus type 1* (HIV-1), which is primarily transmitted through heterosexual intercourse or from mother to child. The risk of being infected with HIV during sexual contact is higher for women than from men, mainly due to the fact that the large surface area of the vagina is highly exposed to the virus. The symptoms of HIV/AIDS are similar in women and men. However, infections of the female reproductive organs may be more frequent and severe in women with HIV infection. Moreover, a causal relationship between vaginal lesions and susceptibility to HIV-1 infection has been confirmed [24].

These premises highlight the need to combine local and systemic therapy aimed at both preventing and treating vaginal diseases, combined with new systemic antiviral and vaccine therapies.

The HIV-1 life cycle is complex, with duration and results that depend on targeted and activated cells [25]. From the 1990s, the treatment of HIV-1 has been focused on the use of inhibitors of reverse transcriptase, as well as on protease inhibitors, which are essential enzymes for HIV-1 life cycle, alone or in association. 

The first class could be further subdivided in nucleoside/nucleotide derivatives (NRTIs) and non-nucleoside compounds (NNRTIs), which are non-competitive inhibitors.

HIV-1 protease inhibitors are the second group of drugs for HIV therapy and the largest group of inhibitors (currently eight compounds are used). 

Other drugs that inhibit different steps of the viral life cycle have been developed, for example, virus cell entry inhibitors, among which enfuvirtide and maraviroc are currently the only approved. A similar mechanism of action is exhibited by natural compounds such as marine algae polysaccharides, chitosan derivatives, or sulfated polysaccharides, but they are not approved as drugs [26]. 

However, as previously mentioned, HIV-1 standard therapy shows some drawbacks, mainly due to resistance development. For this reason, the combination of several antiviral agents has become more and more advantageous. Nevertheless, high toxicity levels are associated to long-term treatment, e.g., renal and hepatotoxicity, diabetes mellitus, and immunodeficiency, which might be reduced by periodic interruption of the treatment interruptions, even if this hypothesis requires further exploration [25]. Moreover, the use of microbicides to prevent rectal and vaginal transmission of HIV-1 is becoming noteworthy.

In this review, we focalized our attention mainly on the development of novel drug delivery systems for vaginal drug administration of anti-HIV-1 compounds.

### 2.3. Fungal Infections

Vulvovaginal candidiasis (VC) is a fungal infection caused by yeasts of *Candida* species that could extend to other female organs. *Candida albicans* is the most isolated pathogen, responsible for 50–70% of systemic fungal infections. Diabetes mellitus, obesity in association with intertrigo due to rubbing and sweating, high estrogen levels (particularly during pregnancy), genetic factors, atopic diathesis, type 1 allergies, and immune suppression are predisposing host factors. On the contrary, there is no connection between the frequency of antibiotic administration and the development of VC. Common treatments are based on the administration of antifungal agents such as imidazole class (ketoconazole, chlortrimazole, fluconozole, etc.), and echinocandins (capsofungin, micafungin, anidulafungin), amphotericin B, and flucytosine, usually administered in association. Allylamines such as terbinafine and naftifine or griseofulvin, but also the orally administered probiotics represent other alternatives to common treatment. However, all traditional antimycotic agents do not have a broad spectrum of action and exhibit high toxicity/low bioavailability, requiring further studies to improve current therapies [27]. 

### 2.4. Parasitic Infections

Trichomoniasis is a sexually transmitted infection considered responsible for high reproductive morbidity and a promoter of both HIV infection and transmission. It is caused by *Trichomonas vaginalis,* an obligate parasite that phagocytizes vaginal epithelial cells, erythrocytes, and bacteria, and is itself ingested by macrophages. At first, *Trichomonas vaginalis* infects the squamous epithelium of the genital tract with a 4–28-day incubation period. Then, the infection can last also for months or even years and can be asymptomatic (up to 50%) but also severe, with serious sequelae, especially in women [28]. Standard treatments consist in 2 g one-dose oral administration of metronidazole or tinidazole. Although both are the most effective drugs for treating *Trichomonas vaginalis* infection, in case of hypersensitivity to 5-nitroimidazole drugs, disulfiram and nithiamide might represent alternatives. However, there are only limited data on alternatives to nitroimidazole derivatives, and thus new antitrichomonal agents or new pharmaceutical formulations are needed. Currently, prevention methods using condoms or local intravaginal formulations remain the most reliable form of protection, but certainly the development of vaccines could be a winning strategy [29].

## 3. Local Drug Delivery Systems for Vaginal Infections: From Conventional to Innovative Strategies

As previously reported, in developing novel vaginal drug delivery systems, the physiological characteristics of the vagina must be accurately considered because they could influence drug dissolution and transport across the membranes. Indeed, like other mucosal delivery systems, when drugs are administered through the vaginal route they are absorbed transcellularly (diffusion through cells), paracellularly (through tight junctions), or vesicularly, or are transported by receptors. Furthermore, changes in vaginal epithelium thickness, composition, volume, viscosity, and pH of vaginal fluid may influence drug absorption either negatively or positively. For example, the absorption of poorly water-soluble drugs could be increased in the presence of high volumes of the vaginal fluid; on the other hand, an increase in its viscosity can represent a barrier to drug absorption. Among biological factors, also enzymes (mainly proteases), enzyme inhibitors, proteins, amino acids, lactic acid, acetic acid, glycerol, urea, glycogen, and ions may affect intravaginal drug formulation stability or drug absorption. In addition, drug properties, e.g., molecular weight, lipophilicity, ionization, and surface charge, are other important parameters that need to be considered. Traditionally, solutions, ovules, foams, gels, and tablets have been used as vaginal formulations, but it was demonstrated that drug distribution varies in a meaningful way with the nature of the formulation. Among all, solutions, suspensions, and foams show better properties compared to tablet forms. 

At the same time, bioadhesive formulations ensure a prolonged residence time in the vaginal cavity. In Table 2, we report some of the most employed commercial vaginal dosage forms [30].

Generally, the rate of drug distribution after intravaginal administration and the ability of the formulation to coat the whole organ are hard to be quantified. Moreover, mucosal surface properties play an important role in influencing biodistribution, retention, and uptake across the mucus. For example, surface chemistry and charge could influence attraction or repulsion between mucin fibers. 

To overcome all these limitations, there is the need to develop novel drug delivery systems on the basis of nanotechnologies to increase drug physical stability and bioactivity, reduce toxicity, and enhance patient compliance. However, particular attention should be paid to the size of these nanosystems. Indeed, particle diameters control the ability to fit within the mucin mesh pores. Moreover, the ability to form aggregates potentially affects the diffusion or the immobilization of the particles across mucus. In fact, particle self-binding could lead to the formation of aggregates immobilized on the surface that exhibit a mucoadhesive behavior. This property improves the residence time of the system, providing its intimate contact with the mucosa; in addition, the concentrations of NPs onto mucus could influence its normal structure causing the collapse of mucin fibers [32].

Nanosystems such as liposomes, nanoparticles (NPs) (including solid lipid NPs, polymeric NPs, or inorganic NPs), and micelles could be exploited to improve local vaginal drug delivery. Different types of biocompatible and biodegradable polymers already approved by the U.S. Food and Drug Administration (FDA) and by other authorities have been proposed for controlled drug delivery at mucosal surfaces. Among these, poly(lactic-*co*-glycolic acid) (PLGA), cellulose derivatives, triblock copolymers of poly(ethylene oxide) (PEO)/poly(propylene oxide) (PPO) (PEO-PPO-PEO), polycaprolactone (PCL), polyacrylates, poly(ethylene glycol) (PEG), poly(vinyl alcohol) (PVA), and alginate have been studied either as additives or as matrices of NPs.

It is well known that the use of PVA as surface stabilizer or the surface coating with PEG chain contributes to vaginal adhesion and promotes the formation of bonding with the hydrophobic regions of mucin, enabling the rapid diffusion in cervicovaginal mucus. However, although the above-mentioned mucoadhesive polymers can promote vaginal drug retention, they could lead to mucus disruption and to the loss of its barrier properties against xenobiotics and pathogens. The intrinsic toxicity of these polymers should also be taken into account, even if in general they are eliminated by mucus turnover and discharge, or by natural cell shedding, when epithelial penetration occurs [33]. An alternative is represented by thiolated hyaluronic acid, preactivated with 6-mercaptonicotinamide, which exhibits enhanced stability and mucoadhesive properties without toxicity, as demonstrated by a study of Nowak et al. (2014) [34]. 

During the last decades, various types of nanosystems have been explored for vaginal drug delivery (mucoadhesive, non-mucoadhesive, or mucus-penetrating) in an attempt to overcome the limitations of conventional dosage forms. The most innovative forms are discussed in the following paragraphs, with a particular attention to the nanotechnology-based antimicrobial strategies.

### 3.1. Vaginal Drug Delivery Systems for Bacterial Infections

#### 3.1.1. Bacterial Vaginosis

**Figure 1:**
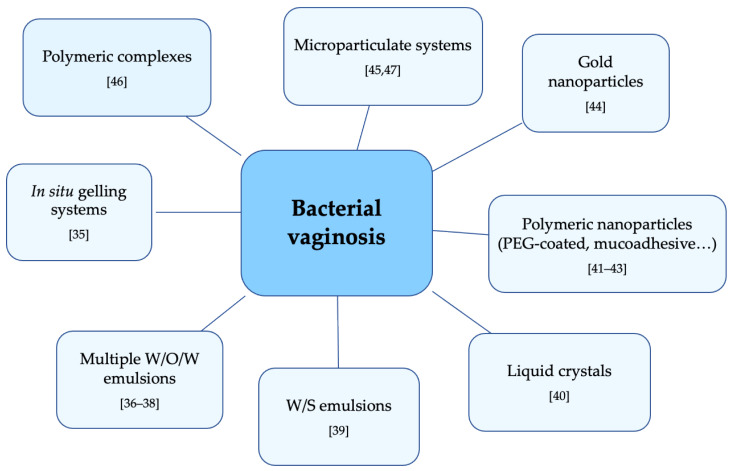
Innovative delivery systems for bacterial vaginosis local treatment.

Vaginal delivery of clindamycin or metronidazole is one of the commonest therapies in the treatment of BV, but its efficacy is not ideal. Several researchers are working to improve existing formulations or to create new dosage forms (Figure 1).

In situ gelling systems such as hydrogels represent a valuable alternative to conventional gels due to their ability to provide the most convenient liquid application in the field of topical delivery and to remain in situ for a prolonged period. The liquid topically applied turns into gels because of physical and/or chemical change such as pH variations, concentration of calcium ions, or temperatures, amongst others. To obtain a sustained release, one can either disperse the drug in the gel or initiate an interaction between the drug and an oil phase included in the gel. 

With this aim, Chopra et al. (2007) designed and evaluated a new polyherbal antimicrobial mucoadhesive drug delivery system using three different grades of Carbopol resins, which showed excellent bioadhesive properties on the mucosa surface [35]. 

Multiple water–oil-in-water (W1/O/W2) emulsions are an alternative system that can entrap substances in the inner aqueous phase, protecting them from the outer environment and modulating their release; moreover, several actives can be located in the different compartments. These systems are easy-to apply formulations with efficient, light, non-greasy, and non-sticky textures that may facilitate the application and prevent irritation and burning in the vulvovaginal region [36,37]. Özer et al., in 2007, formulated two multiple emulsions containing metronidazole and ornidazole in different aqueous phases, monitoring both drug release in acidic/alkaline medium to understand the role played by pH on the release. A proper release was observed in alkaline medium, which is convenient for vaginal infection, and both the systems were found to be locally effective in the vagina [38].

In addition, other innovative emulsions have been recently investigated for local drug administration in vaginal diseases. Mucoadhesive water in silicone emulsions (W/S emulsions) can resist the clearance effect of vaginal fluid by promoting the formation of a highly resistant adhesive layer. This characteristic makes W/S emulsions a good alternative to vaginal hydrogels, whose rapid swelling in the aqueous medium determines loss of consistence and bioadhesion. Moreover, W/S emulsions have good rheological, mechanical, and organoleptic properties; high biocompatibility; and low toxicity. For these reasons, Campaña-Seoane et al. (2019) studied the in vivo behavior and drug absorption/distribution to the sexual organs of a W/S mucoadhesive emulsion containing ciprofloxacin, which is a broad-spectrum antibiotic frequently used in the treatment of sexual infections. As expected, the system exhibited an excellent in vivo bioadhesion and high resistance to vaginal clearance with a constant ciprofloxacin release during at least 6 h, which showed W/S emulsions to be a potential tool for the treatment of sexual tissue infections [39].

Lastly, liquid crystals represent an emerging class of drug delivery systems constituted by polar lipids that can reorganize themselves into 3D structures in water. Liquid crystals have a malleable structure that could be controlled to allow both sustained drug release and ease of administration. Recently, some authors (2019) evaluated the controlled delivery into cells of ciprofloxacin encapsulated in liquid crystals, demonstrating that they significantly enhance bacterial killing compared to free drug formulation and highlighting their potential use for the treatment of FRT infections and/or topical administration for the upper infections [40].

Besides all these platforms developed to increase active agents’ delivery and retention time in the FRT for the treatment of BV, in recent years, NPs have gained increasing attention.

Mucoadhesive multiparticulate systems exhibit several advantages for vaginal administration compared to other commonly used dosage forms because they can cover a wider mucosa area and allow a prolonged drug permanence on its surface and a sustained release over a long period of time. They offer several advantages such as drug protection from degradation in the acidic vaginal microenvironment, sustained release, high entrapment, and localized delivery of both hydrophobic and hydrophilic agents. Polymeric NPs, with their small sizes (from 70 to 300 nm), exhibit good physical properties and are suitable for the delivery to the FRT. Surface modifications, e.g., ligands conjugation or adsorption, improve retention, diffusion, and cell-specific targeting. Polymeric NPs are made of non-toxic and non-inflammatory polymers, making them biocompatible systems [41]. In a recent work by Marciello et al. (2019), chitosan NPs encapsulated in hydrophilic freeze-dried cylinders were developed for the delivery of a peptide drug. They were assessed to improve the peptide adhesion to the vaginal mucosal epithelium. In addition, hydrophilic freeze-dried cylinders enabled the application and the quick release of NPs into the vaginal zone after the contact with the aqueous vaginal medium, promoting peptide penetration inside vaginal mucosa [42]. 

However, although several NPs show greater mucoadhesivity than conventional systems, they hardly overcome the mucus barrier requiring high administration doses to reach the underlying tissues. A promising approach overcoming this drawback seems to derive from “stealth” NPs. In a study by Sims et al. (2019), stealth PEG-coated NPs exhibited appropriate mucus-penetrating properties as well as improved drug retention and transport. Tests performed on PLGA NPs showed that prolonged retention profiles for 24 h were obtained with the greatest percentage of PEG coating (5, 8, 25%) [43]. 

Lastly, NPs could also be exploited for rapid diagnosis of vaginal infections. With the aim of overcoming the high costs and time-consuming nature of diagnosis laboratory methods, Hashemi et al. (2019) developed gold NPs conjugated with antibodies to be used in an agglutination test for the detection of vaginal infections. Results demonstrated that gold NP tests are simple, accurate, and cost-effective, being a potential way to detect vaginal infections [44].

Another example of multiparticulate systems is represented by chitosan–alginate microspheres such as those formulated by Maestrelli et al. (2018) for the vaginal administration of cefixime, used in order to overcome drawbacks following its oral administration. The effect of increasing drug loading amount was investigated; mucoadhesion studies reported that all formulations were able to remain in situ for more than 2 h (on excised porcine vaginal mucosa), showing good adhesion properties. Entrapment efficiency grew by increasing the starting loading drug concentration, reaching a plateau at the optimal drug-to-polymer ratio. Microbiological studies confirmed the potential advantages of such systems that can be administered inside a capsule by a commercially available vaginal applicator. After capsule dissolution, the distribution of chitosan–alginate microspheres occurs with a prolonged in situ activity of cefixime due to their good mucoadhesion and extended release properties [45]. 

Chitosan/alginate can also be used for the development of complexes through the mixing of polymeric solutions with different chitosan/alginate molar ratios and freeze-drying the precipitates. They have been used to prepare chlorhexidine-loaded vaginal inserts. Drug distribution; mucoadhesion; and in vitro water uptake, release, and microbiological tests demonstrated that these systems can overcome messiness and leakage of formulations, increasing patient compliance. Moreover, the chlorhexidine digluconate release from this insert can inhibit the pathogenic agents responsible for vaginitis [46].

Lastly, multiparticulate systems also include microbeads, such as those prepared by Szymanska et al. (2019) via spray-drying method. Zidovudine, a water-soluble antiretroviral agent, was selected as a potential active agent to be applied as vaginal microbicide. Rheological behavior, spreadability upon dilution, and drug dissolution profile were evaluated in vitro in a simulant vaginal fluid. In a second step, cytotoxicity was assessed on human vaginal mucosa cell line VK2/E6E7. An initial rapid/moderate burst effect was observed in formulations spray-dried at 120 °C and 160 °C, followed by a prolonged, swelling-dependent drug release stage. The inlet spray-drying temperature is an important factor since, through changing it, the swelling capacity and in vitro drug release of microbeads could be easily modified. Moreover, zidovudine-loaded systems were able to reduce cell viability after 24 h of incubation, being shown to be a promising microbicide delivery platform for the delivery of water-soluble drugs into the vaginal environment [47].

#### 3.1.2. Chlamydia

NPs have gained increasing interest in the treatment of *Chlamydia trachomatis*. Metal-based NPs, for example, exhibit antibacterial activity due to their ability to produce reactive oxygen species damaging the bacteria and to bind to RNA or DNA, thereby hindering microbial replication processes. Among all, silver NPs are the most diffused and described in literature. They can penetrate bacterial cell walls, damaging the structure of cell walls and resulting in cell death by producing free radicals or interacting with the thiol groups of many bacteria vital enzymes. Moreover, they can interact with the phosphorus and sulfur atoms of DNA, inhibiting DNA replication of the bacteria. NP shape and size have been reported to influence the antibacterial effects. In fact, smaller nanosized silver particles seem to be more effective than larger ones, and spherical shaped NPs exhibit enhanced antibacterial activity in comparison with the triangle-shaped forms [48]. NPs could also be used as a strategy to reduce vaginal infection of *Chlamydia trachomatis*. 

A pilot study by Sangare et al. (1999) evaluated the in vitro anti-chlamydial activities of liposome-encapsulated doxycycline and tetracycline in comparison with free drugs. Drug loaded cationic, anionic, and neutral liposomes were prepared by sonication, showing a higher inhibitory effect on *Chlamydia trachomatis* growth than free doxycycline and tetracycline. These results demonstrated that developed liposomal formulations can be very useful as an alternative strategy for antimicrobial therapy [49]. 

More recently, Yang et al. (2017) [50] developed a novel combination therapy consisting of siRNA-polyethylenimine-encapsulated NPs made of PLGA-PEG that are able to knock down the expression of platelet-derived growth factor receptor-β (PDGFR-β) and simultaneously induce autophagy through the encapsulation of a cationic polymer. In this way, systemic adverse reactions and resistance caused by antibiotic oral administration can be avoided. The use of PDGFR-β siRNA-PLGA-PEG NPs should start days before sexual interactions due to the delayed onset of action of gene silencing by PDGFR-β siRNA. Its use may continue in the long term since the gene knockdown effect of siRNA is temporary and because the continuous intracellular level of siRNA needs to be maintained in order to obtain sustained gene knockdown. However, the duration of administration and dosing frequency must be determined by future in vivo and clinical studies. Moreover, in the future, other potential receptors can be targeted simultaneously to enhance the prevention efficacy. An interesting study found in the literature [51] reports the use of PLA-PEG NPs for the sustained delivery of a *Chlamydia trachomatis* recombinant peptide to increase systemic adaptive immune responses that are required by a vaccine candidate. Results highlight PLA-PEG’s potential for vaccines due to the slow release and potentiated effects to bolster immune response.

In a recent patent, folic acid- and/or fluorescence-labelled PLA-PEG NPs for imaging chlamydia infection were described, wherein NPs targeted folic acid receptor-expressing cells infected with chlamydia. In an imaging experiment, folic acid-conjugated NPs accumulated in significantly high amounts in the genital tract [52].

#### 3.1.3. Gonorrhea and Syphilis

The growing antibiotic resistance against gonorrhea emphasizes the need to identify new antimicrobials. Chitosan has shown a wide spectrum of antimicrobial activity against Gram-positive and Gram-negative bacteria. Its antimicrobial activity has been demonstrated by different theories, but the exact mechanisms are still unknown. This suggests that there is a potential for chitosan NPs as an antimicrobial agent against pathogens such as gonococcus. In a study by Alqahtani et al. (2020) [53], chitosan NPs were formulated and then characterized through examining their antimicrobial activity against *Neisseria gonorrhoeae*. They revealed an anti-gonococcal activity against the tested strains, which took place at specific concentrations, at which NPs reduced bacterial adhesion to cells and were cytocompatible, confirming the therapeutic potential of chitosan NPs against *Neisseria gonorrhoeae*.

In another study (2019), chitosan-based liposomes-in-hydrogel loaded with polyphenols (resveratrol and epicatechin) were developed. Each polyphenol was singularly incorporated into the formulation. It appeared that when liposomes were incorporated into chitosan hydrogel, the gel’s texture properties could be improved. In addition, the developed systems displayed adequate mucoadhesive properties, achieving a prolonged retention time; indeed, a prolonged and controlled release of the entrapped polyphenols was reached. These formulations did not show direct toxic effect to living cells at considerable treatment concentrations. Epicatechin showed enhanced anti-oxidative and anti-inflammatory activity at the highest concentrations when formulated in the liposomes-in-hydrogel system. Moreover, chitosan helps the biological activities, making the developed system a potential vaginal delivery platform for the treatment of localized infections and inflammations [54]. 

Finally, the encapsulation of octylglycerol into liposomes exerted an increased efficacy against *Neisseria gonorrhoeae* compared to a conventional gel formulation. The efficacy was maintained for over two months. Moreover, no toxicity was observed for the octylglycerol-loaded liposomal formulation when tested ex vivo in a human ectocervical tissue model or in vivo in a macaque model [55].

**Figure 2:**
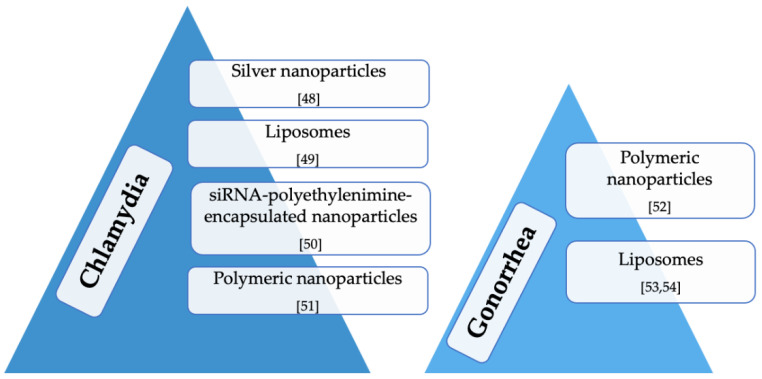
Innovative drug delivery systems for local treatment of chlamydia and gonorrhea.

Figure 2 summarizes the recently developed drug delivery systems for the local treatment of chlamydia and gonorrhea.

As for syphilis, penicillin is the first-line treatment option; in particular, benzathine penicillin G provides a convenient regimen in terms of ease of administration and with demonstrated anti-treponemal activity. However, syphilis is an important and increasing public health problem for which timely diagnosis is important to limit its clinical effects. Recently, a novel quantum dot-based point of care test for syphilis has been developed [56]. Moreover, a Goldmag immune probe, as part of a NP-based colorimetric assay for the detection of syphilis, has also been proposed [57]. In addition, tests have demonstrated that polyelectrolyte-coated gold magnetic NPs are sensitive and selective when used in a syphilis immunoassay, suggesting a possible use in point of care diagnostics for syphilis screening [58].

Therefore, syphilis provides an example of potential applications of nanomedicine not only in the treatment but also in the diagnosis and prevention of vaginal infection diseases. 

### 3.2. Vaginal Drug Delivery Systems for Viral Infections

**Figure 3:**
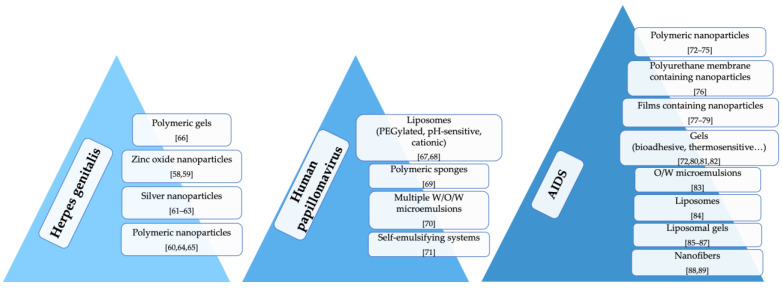
Innovative drug delivery systems for the local treatment of viral vaginal infections.

Approved therapies for viral vaginal infections are based on the use of inhibitors of the viral DNA replication. Although they are effective against primary infections, they cannot protect against the viruses in the latent state, resulting in the reoccurrence of the diseases. Thus, there is a considerable interest in discovering novel therapeutic approaches preferentially acting through different mechanisms from those of the conventional approaches (Figure 3). 

#### 3.2.1. Herpes Genitalis

Currently, either NPs or NP composites are under study to control HSV-2 infections. As an example, zinc oxide NPs have been found to exhibit significant antiviral activity in both the in vitro and in vivo mice model of HSV-2 infections. In a study by Antoine et al. (2012), three different treatment modes were used: prophylaxis, therapeutic, and neutralization. In all of the treatment modes, zinc oxide NPs showed promising results in blocking HSV-2 infections [59,60]. Another study showed the possibility of using mucus-penetrating NPs as antiviral agents—acyclovir monophosphate-loaded mucus-penetrating NPs showed an increased drug retention and distribution, thus providing an effective protection against HSV-2 challenge [61].

The anti-bacterial and antifungal properties of silver NPs have been also widely studied. Moreover, tannic acid exhibits antioxidative, anticancer, and antimicrobial properties, preventing virus attachment, entry, and cell-to-cell spread, as reported by some studies. Orlowski et al. (2014) developed tannic acid-modified silver NPs with good antiviral properties both in vitro and in vivo by direct blocking of viral attachment and penetration. Altogether, the authors showed that tannic acid-modified silver NPs represents a potential microbicide for HSV infection in the mucosal tissues [62]. The effective anti-viral properties with additional adjuvant properties boosted the anti-viral response, not only during the primary infection, but also later upon recurrent infections [63]. In fact, silver NPs at nontoxic concentrations can inhibit HSV-2 replication when administered prior to viral infection or soon after initial virus exposure, suggesting that their mode of action occurs during the early viral replication phases [64]. 

Other studies involved the development of drug-loaded PLGA NPs for the intravaginal administration of siRNA for the prevention of genital HSV-2 infections in mice. PLGA NPs were designed to interfere with HSV-2 infection by siRNA-mediated knockdown of nectin, a host cell protein. In this way, the survival of mice, inoculated intravaginally with a lethal dose of HSV-2 and treated with PLGA NPs, increased considerably to more than 28 days compared to the untreated mice. Furthermore, this system provides a safe delivery platform that employs materials that are already approved by the FDA and that can be modified to improve delivery of other microbicides [65]. In another study, ftibamzone-loaded NPs significantly enhanced the solubility and absorption of ftibamzone compared to free drug and had the potential to be used as an effective delivery system to treat genital herpes [66]. 

A controlled release could also be reached using in situ gelling systems loaded with polymeric NPs containing acyclovir. These Pluronic F127 gels exhibited enhanced permeability through vaginal membrane. Moreover, NPs showed improved viability for vaginal epithelial cell lines. Significant enhancements in drug mean residence time and in the relative bioavailability were observed in comparison with those of the pure drug. A sustained in vivo drug release was demonstrated, ensuring the stability of the formulation for clinical therapy in the female population [67].

#### 3.2.2. Human Papillomavirus (HPV)

Due to their favorable characteristics, the use of liposomes as drug carrier represents a possible tool for the treatment of HPV. In a study published in 2016, liposomes entrapping interferon alpha-2b were PEGylated to allow them to penetrate mucus for a localized action against HPV vaginal infections. The release rate performed in vitro on a model membrane showed a negligible release of interferon alpha-2b from both the control and the liposomal formulation. However, the experiments performed ex vivo on the vaginal tissue collected from pregnant sheep highlighted the positive effect of PEGylation on interferon alpha-2b mucus penetration. Moreover, the absence of interaction between the PEGylated liposomes and mucin was also demonstrated, confirming this system as an interesting strategy to improve the efficacy of vaginal anti-viral therapy [68].

As previously mentioned, human cervical cancer can be induced by the expression of particular types of HPV oncogenes. Thus, to knockdown the sequence-specific genes is deemed a novel therapeutic approach to contrast the human cervical cancer. 

As an example, in 2020, Zhen et al. developed pH-sensitive cationic liposomes as safe and efficient carriers of CRISPR/Cas9, a gene-editing agent able to correct errors in the genome to cancer tissues. In vivo experiments in mice showed that these nanoliposomes significantly inhibited tumor growth without toxicity. Therefore, this system can be considered a valuable delivery carrier of nonviral gene editing elements, offering a promising route to precision medicine-based cancer therapeutics [69]. 

Another interesting approach, proposed by Furst et al. (2016), is the delivery of siRNA to the vaginal tissues by means of a polymeric solid mucoadhesive system, loaded with muco-inert PEGylated lipoplexes, able to be rehydrated by the vaginal fluids to form a hydrogel. The obtained solid systems (sponges) exhibited adequate characteristics such as hardness, deformability, and mucoadhesive properties. However, further studies are required to validate active siRNA sustained release [70].

Wang et al. (2019) designed a thermosensitive gel obtained from W1/O/W2 multiple microemulsion and exploited it for siRNA delivery [71]. The microgels obtained by self-emulsification of multiple microemulsions provided higher mucosal residence time and greater exposure. Moreover, the W1/O/W2 system helped siRNA transport through the bio-membrane and increased transfection efficiency due to improved in vitro/in vivo stability and enhanced endocytosis. 

Finally, some studies [72] reported the potential of a vaginal curcumin cream to eradicate HPV infection, but a drawback is represented by the strong interaction between curcumin and mucus, which lead to a reduced spread over the mucosa. To overcome this shortcoming, self-emulsifying drug delivery systems offer an attractive choice for curcumin vaginal delivery as they confer high mucus permeation and rapid spreading of curcumin on the vaginal mucosa. Results showed that compared to conventional O/W creams, self-emulsifying systems are advantageous since they promote curcumin permeation through the mucus gel layer. For these reasons, they could be proposed for many drugs, which in this way could extend their therapeutic efficacy after vaginal application. 

#### 3.2.3. AIDS 

AIDS is a sexually transmitted infection that differs from the previously discussed vaginal infections because vaginal tissues are not targets of HIV disease but actively participate in its pathogenesis. Furthermore, there is strong evidence of a relationship between vaginitis and HIV acquisition.

The last years saw significant progress in the formulations of carrier systems suitable for the prevention and treatment of HIV infection. 

One strategy is represented by the use of nanosystems, having intrinsic antiviral activity or acting as carriers for antimicrobials. In particular, polymeric NPs were shown to be able to enhance mucosal distribution and retention of many antiretroviral compounds [73]. As an example, NPs made of biodegradable and non-toxic PLGA, loaded with efavirenz or saquinavir, exhibited in vitro a strong protection against HIV-1 BaL infection and inhibitory effects significantly greater than free drug. 

A study by Chaowanachan et al. (2013) also demonstrated that the combination of tenofovir with either efavirenz-loaded or saquinavir-loaded NPs synergized combined drug effects, emphasizing the potential of NPs in realizing unique drug–drug activities [74]. 

Polymeric NPs could also be functionalized through surface coating, as in the study of Das Neves et al. (2013), in which dapivirine-loaded PCL NPs coated with PEO (PEO-PCL-NPs), sodium lauryl sulfate, or cetyl trimethylammonium bromide were developed. Test of permeability and retention carried out in cell monolayers and pig vaginal mucosa showed different results according to the coating type. Overall, PEO-PCL-NPs were the most effective in increasing microbicide drug residence time at epithelial cell lines/mucosal tissues [75]. 

New therapeutic alternatives against HIV-1 diffusion are based on peptides able to inhibit the HIV-1 fusion in target cells at an early stage. However, such peptides exhibit some drawbacks, e.g., short half-life, rapid clearance, low bioavailability, and poor ability to cross the physiological barriers, making them unattractive for the pharmaceutical industry. To overcome these limits, Ariza-Sáenz et al. (2018) developed glycol chitosan-coated PLGA NPs, exploiting their ability to incorporate an HIV-1 inhibitor peptide, allowing its release into the vaginal epithelium. The results confirmed that these NPs are able to permeate vaginal tissue crossing the vaginal fluid to reach the epithelium and release the peptide inside, allowing a more easily permeation of HIV-1 inhibitor than the free peptide [76].

However, although nanocarriers are beneficial for the intravaginal delivery of anti-HIV drugs and siRNA, a continuous release of drugs is not desirable for the prevention of HIV. In this case, it is preferred that the release from the nanocarrier occurs only during sexual intercourse in order to avoid unnecessary exposure to the drug and to reduce side effects. For this purpose, Kim et al. (2018) developed a pH-responsive interconnected porous polyurethane membrane for intravaginal release of NPs containing acid-labile therapeutic agents such as siRNA. As a result, drug release was close to zero at pH 4.5 (normal pH of the female genital tract), while at pH 7.0, a significant increment of drug release was observed. Moreover, the electrospun porous pH-responsive polyurethane membrane is nontoxic for human vaginal epithelial cell line (VK2/E6E7) and human T-cell line and does not induce inflammation, being a novel biomaterial for “smart” intravaginal delivery of anti-HIV drug-loaded NPs [77]. 

In the field of drug delivery, films comprise thin, flexible, soft, and often translucent polymeric solid strips, in which active ingredients are dissolved or dispersed. After administration and contact with vaginal fluids, films typically dissolve, decrease their volume, and become a gel-like fluid. However, films can also be designed to slowly disintegrate. They exhibit well-documented characteristics such as the ability to co-formulate multiple drugs, good stability upon storage, and high acceptability by women since films are portable, easy to administer without the need of an applicator, and are known to produce minimal vaginal leakage. 

The application of films as platforms for vaginal administration of nanosystems, namely, drug-loaded polymeric NPs, is recent, due to the ability of the tight polymeric matrix that surrounds nanosystems to limit phenomena such as particle aggregation and segregation, leading to colloidal instability [73]. In a study by Cunha-Reis et al. (2016), for example, efavirenz-loaded and tenofovir-loaded PLGA NPs were incorporated into fast-dissolving films. In vivo studies revealed that the association between NPs and film contributed to enhance drug retention in vaginal tissue, mainly during the first hours after administration. The presence of both tenofovir and efavirenz at the systemic level was low, helping to prevent adverse effects and potential toxicity. The NPs-in-film system was also safe for vaginal administration, even if further studies are required [78]. A similar study was carried out by Machado et al. (2016). In this case, tenofovir-loaded PLGA/stearylamine composite NPs were incorporated into a hydroxypropyl methylcellulose/PVA-based film, resulting in a tenofovir release in a biphasic fashion (approximately 30% in 15 min, followed by a sustained drug release for 24 h). Moreover, the incorporation of NPs further improved the adhesive properties of the film, even if cytotoxicity of both NPs and film was significantly increased by the incorporation of stearylamine, still remaining at tolerable levels for tenofovir vaginal delivery [79]. Moreover, comparing single or double-layered films containing free drug with drug loaded NPs incorporated into double-layered films resulted in double-layered films significantly reducing drug burst effect as compared to fast releasing single-layered films. Furthermore, drug release delay was noticeable when drug-loaded NPs were incorporated into double-layered films, being an interesting approach to sustain the release of anti-HIV drugs [80]. 

Gels are the most popular dosage forms used for vaginal drug delivery, mainly due to their technological acceptability by users, versatility, and low production costs. Carbomers and hydroxyethyl cellulose are the most commonly gelling agents used [73]. As an example, Martín-Illana et al. (2019) recently developed vaginal bioadhesive emulgels and/or bigels to prevent sexual transmission of HIV through the controlled release of tenofovir [81]. 

Moreover, several studies reported the use of thermosensitive hydrogels based on chitosan and glycerophosphate, even though the burst release of loaded drugs is becoming a critical challenge when these types of hydrogels are employed as a drug delivery system. To overcome this problem, chitosan-glycerophosphate hydrogels containing tenofovir and *Bletilla striata* polysaccharide microspheres were developed by Yang et al. In this way, microspheres could be localized around the administrating target because the hydrogel can transform itself into semisolid gel after administration. Then, the system continually releases the drug from the double-component formulation, further extending its releasing time. *Bletilla striata* polysaccharide was co-delivered with tenofovir due to its antibacterial, antitumor, and hemostasis properties and its ability to protect gastric mucosa and to promote wound healing. Such thermosensitive chitosan hydrogels containing polymeric microspheres exhibit the potential to be an appropriate formulation for sustained-release vaginal delivery system [82]. 

Similarly, Tian et al. proposed a hybrid thermosensitive hydrogel based on nanosized layered double hydroxides (LDH) and poloxamer. LDH are clay-based NPs that exhibit high biocompatibility and enhanced drug delivery efficiency. The aim of the authors was to load into this hybrid hydrogel both hydrophilic theaflavin and hydrophobic nifeviroc, dispersing theaflavin into poloxamer solution and intercalating nifeviroc into LDH, forming a complex hydrogel-based microbicide candidate for blocking HIV entry into cells. In vivo studies demonstrated that this system undergoes rapid sol–gel transition at body temperature, exhibiting poor mucosal irritation and sustained controlled release of both hydrophilic and hydrophobic drugs into the blood. Moreover, the local concentration of both drugs is hundreds times of that observed in blood, promoting its application as a pre-exposure prophylaxis microbicide during sexual behavior [83]. 

An increased adhesion and retention time in the vaginal mucosa could also be obtained by adding a gelling polymer to an O/W microemulsion, as in study by Mirani et al. (2019). This microemulsion gel was loaded with a curcumin derivative explored for the prophylaxis of HIV-1 infection. Compared to free drug, the developed system exhibited a coitus-independent release profile, rapid time-independent intracellular uptake, and fourfold increase in efficacy, making it a potential candidate capable of prevention of HIV-1 infection associated with unprotected sexual intercourse [84]. 

As already reported, liposomes have been investigated as a potential vaginal delivery system, but the liquid nature of the preparation represents a meaningful disadvantage. The addition of mucoadhesive and polymeric agents that lead to the formation of liposomal gel formulation could be a promising strategy. In this way, HIV-1 envelope glycoprotein CN54gp140 could be administered as HIV-1 vaccine through its encapsulation within neutral, positively, or negatively charged liposomes, as reported in a study by Gupta et al. (2012). These vesicles were then incorporated into hydroxyethyl cellulose aqueous gel exhibiting good encapsulation efficiency and suitable mucoadhesive strength. The possibility to be lyophilized makes this liposomal gel a stable and practical dosage form suitable for vaginal application [85]. 

Liposomal hydrogels were also used for the delivery of an interesting drug combination based on tenofovir and emtricitabine, which demonstrated an interesting co-action in pre-exposure prophylaxis. Due to the yield strong impairment at the polar head groups of lipid bilayers exhibited by emtricitabine, the final formulation developed by Faria et al. (2019) consisted of liposomes loaded with tenofovir further incorporated in carbomer-based hydrogels with emtricitabine. Limitations as leakage and messiness can usually be minimized by simply using lower volumes of gel per administration [86]. Moreover, compared to emulsions, liposomal gels allow higher drug incorporation, are stable upon storage, and are well-tolerated and non-irritating after vaginal administration. In addition, drugs are more absorbed from liposomal formulations than from emulsions or gels, which may be advantageous when systemic absorption after vaginal administration is required [87,88].

Nanofibers are a solid dosage form that could help to overcome messiness and leakage characteristics of liquid or gel-based dosage forms. In particular, electrospun nanofibers can incorporate a remarkable drug amount, up to 60% by mass, and their polymer ratios and core-shell structure can be modified to control drug release over time for days or weeks. Moreover, nanofibers exhibit the ability to encapsulate different agents, show flexibility in processing parameters, and have multiple conceptual geometries to achieve practical and user-friendly administration. Indeed, Krogstad and Woodrow (2014) hypothesized that PVA fibers might be a good candidate for a quick-dissolving peri-coital microbicide due to the amorphous domains of PVA, which allow for swelling and dissolution in water, and the large surface area, which may further promote fast dissolution and drug release. Using only water as a solvent, 60% wt/wt tenofovir was encapsulated into electrospun fibers without compromising fiber integrity. The results revealed that high crystallinity of drug solid dispersion may not significantly modify release kinetics of tenofovir from electospun fibers, but it may influence biodistribution by reducing drug solubility. However, nanofibers’ favorable characteristics support the potential for scale-up of tenofovir-loaded fibers [89]. 

The positive properties of nanofibers could also be exploited to overcome some NP challenges such as poor retention and extensive leakage. In a recent study (2017), Krogstad et al. developed a NP-releasing nanofiber delivery platform, tailoring the two components in order to obtain optimal interactions with mucus. Mucoadhesive fiber for better retention in the vaginal tract and PEGylated PLGA NPs that diffuse quickly through mucus were designed, with PVA and PVP chosen as mucoadhesive polymers. To demonstrate the utility of this dual system for HIV prevention, the authors also compared the pharmacokinetics of etravirine from NP–nanofiber composites with those from aqueous suspensions. Actually, NP–nanofibers composites significantly enhanced both NPs and drug retention in the reproductive tract compared to the aqueous suspensions; in addition, they ensured a sustained drug release up to three days after a single administration, overcoming a major challenge in vaginal delivery of anti-HIV drugs [90].

### 3.3. Vaginal Drug Delivery Systems for Fungal Infections

#### Vulvovaginal Candidiasis

**Figure 4:**
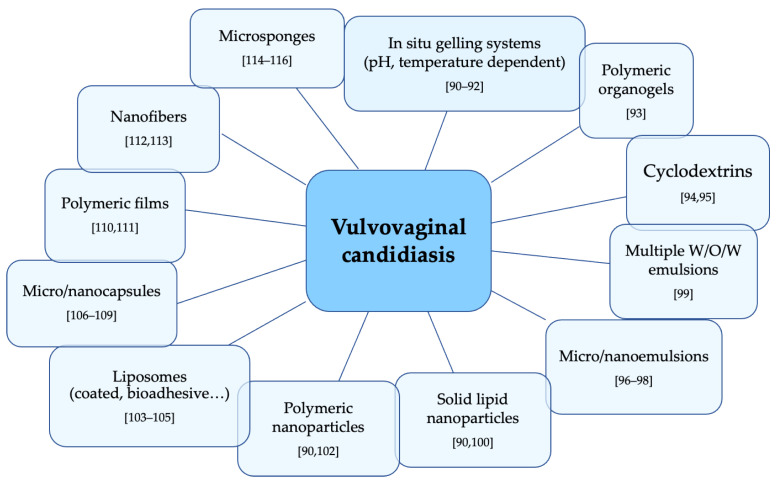
Innovative drug delivery systems for the local treatment of vulvovaginal candidiasis.

In the last years, novel approaches have been explored for the delivery of antifungal agents—most significantly, vesicular drug delivery systems, nano-particulate-based carriers, and in situ gelling systems (Figure 4). They offer several advantages such as targeted delivery, drug protection from degradation, and better drug solubilization and bioavailability. Moreover, these innovative systems act as a depot system, prolonging drug release over time with the advantage of dose reduction and increase of patient compliance. 

As already reported, in situ gelling systems refer to a class of gels in which stimuli such as change in pH, change in temperature, or change in solvent induce the gel formation [91]. Indeed, before body administration, they are in *sol* form, but when administered, they undergo gelation in situ, forming a gel from which drugs are released in a sustained and controlled manner. In situ gels represent an attractive system for bioactive molecules, due to the similarity between their physical properties and those of living tissue that is high in water content, is soft and rubbery in consistency, and has low interfacial tension with water/biological fluids. Thanks to their properties, they could increase patient compliance and exhibit good stability and biocompatibility. Moreover, the production of these devices is quite easy and requires a low manufacturing cost. 

For these reasons, in situ gelling systems can be considered a valuable topical dosage form for the treatment of vaginal infections. With this aim, in 2019, Patel et al. developed and characterized pH/temperature-dependent in situ vaginal gel loaded with clotrimazole. The results showed that all developed formulations exhibited a gelation temperature between 27 and 35 °C. This value decreased with the increment of mucoadhesive polymer concentration, and the nature of the formed gel depends on the concentration of the polymers used. All the tested samples had gelling time in the 2–34 seconds range. In summary, developed systems were simple and easy to use, syringable, allowed easy insertion of gel formulation into the vagina, and were able to release clotrimazole in a controlled way for a prolonged period of time [92]. 

The incorporation of nanomaterials in hydrogels is an innovative approach to obtain hydrogel systems with additional properties recently exploited by some researchers for the vaginal delivery of drugs.

In a recent study, Ci et al. [93] proposed amphotericin B nanosuspension-loaded thermogel as a strategy to satisfy both drug loading and drug diffusion into vaginal mucus. The system containing Poloxamer P407/P188 was characterized by thermosensitivity, high drug loading, and sustained drug release. This formulation resulted in a gelation temperature around 31 °C. In this way, the system was a *sol* during vaginal administration and turned to a gel in situ at body temperature. Moreover, the system was stable during storage and was easy for quality control and large-scale production, allowing a sustained release for about 12 h. The in vivo anti-*Candida albicans* assay confirmed that the antifungal efficiency of amphotericin B nanosuspension-loaded thermogel was better than that of commercial amphotericin conventional forms, being promising for the development of other hydrophobic antifungal drug-loaded formulations. 

As poloxamers have high aqueous solubility, fast dissolution, and unstable rheological properties, it is necessary that the association of poloxamers with other polymers such as sodium alginate are able to improve the mechanical and mucoadhesive properties of thermosensitive gels. For this reason, Querobino et al. prepared voriconazole organogel systems based on the incorporation of an oil phase in a poloxamer (Pluronic F127 and F68, alone or in association) sodium alginate aqueous phase. All the formulations exhibited thermosensitive behavior and the addition of sodium alginate influenced drug release in a concentration-dependent way. Organogels developed with Pluronic F127 and F68 association showed good antifungal activity and low cytotoxicity, being a potential new tool for future vaginal application [94].

Most of the antifungal agents (including voriconazole) are slightly water soluble and poorly stable in aqueous solution. It seems that the use of inclusion complexes of drug with hydroxypropyl-beta-cyclodextrin could overcome these limits. It is also reported that these complexes can increase drug permeation through skin and mucosal layers. In the literature, a study is reported (2019) in which Deshkar et al. prepared voriconazole–hydroxypropyl-beta-cyclodextrin inclusion complex to improve aqueous solubility and stability of voriconazole. This inclusion complex was then incorporated into in situ gelling systems made of poloxamers. Moreover, the effect of the addition of various co-adhesive polymers such as hydroxypropyl methyl cellulose, hydroxyethyl cellulose, and carrageenan was investigated. These developed systems showed good mucoadhesive strength, sustained drug release (more than 8 h), and higher in vivo vaginal tissue uptake/drug dispersion in rats compared to conventional in situ gel, confirming the potential use of such carriers for effective drug delivery [95]. In a similar work (2019), a multifunctional carrier for ketoconazole, obtained via encapsulation of ketoconazole/β-cyclodextrin co-ground mixture into chitosan/gellan gum gel flakes, was developed. In a second step, the developed gel flakes were loaded into an in situ gel of Pluronic F127. The obtained system resulted in a free-flowing liquid at administration time, enhancing the spreading and coating of all the vaginal mucosa, and immediately converted into gel in response to vaginal temperature. Ketoconazole solubility and penetration capacity were also improved. Noteworthy is the possibility to use lower dose regimen of ketoconazole, which improves patient compliance and reduces undesirable side effects [96]. 

Recently, lecithin microemulsion-based lipogels have gained more attention due to their unique characteristics. Their matrix is composed of lecithin, which acts as a surfactant and as a gelling agent in the presence of a nonpolar organic solvent as external phase and a polar agent such as water. These microemulsion gels are thermodynamically stable and biocompatible, offering a superior resistance to microbial contamination. They have low cost of preparation, have higher loading efficiency and storage stability, and do not require organic solvents. Moreover, lecithin microemulsion-based lipogels could solubilize both hydrophilic and hydrophobic drugs and exhibit better performance than liposomal gels, e.g., safety and non-irritant potential, when topically applied. However, more studies are required to investigate their potential ability to eradicate VC in vivo [97]. 

Nanoemulsions are nanometric dispersions of two immiscible phases that are capable of solubilizing lipophilic and hydrophilic drugs, which provide several advantages such as reduced droplet size, large surface area, good tissue contacts, high drug permeation into the tissue, and easy/low-cost preparation. Soriano-Ruiz et al. developed clotrimazole-loaded nanoemulsion to increase drug permeation through mucosae and achieve high and prolonged levels of clotrimazole for an extended period, avoiding the bloodstream and reducing side effects. In actuality, clotrimazole-loaded nanoemulsion seems to provide higher deposition on mucosae and antifungal efficacy compared to commercially available clotrimazole formulations [98]. Nanoemulsions could also be used as a carrier for amphotericin B, providing increased drug release and effective therapeutic potential in comparison with free drug solution and commercial products. Furthermore, this nanocarrier can facilitate the permeation flux and increase drug permeation rate and deposition [99]. 

A similar system is represented by multiple W1/O/W2 emulsions, which can be formed by phase inversion of a simple emulsion and that are of high interest due to their structures and properties, e.g., protection of the included drug and ability to provide sustained release. Nevertheless, generally multiple emulsions are characterized by low stability that can be increased by a combination of polymeric and low lipophilic co-emulsifiers. In a research article published in 2016, multiple W1/O/W2 emulsions have been proposed as innovative and stable carriers for imidazolic antifungal drugs, e.g., clotrimazole [100]. 

Solid lipid NPs are sub-micron range colloidal carriers made of a biocompatible lipid dispersed in water or in an aqueous buffered solution. Controlled and targeted drug release, increased stability, high drug entrapment, easy scale-up, and biocompatibility are some of the advances provided by solid lipid NPs [91]. Some studies report the possibility to enhance antifungal drugs bioavailability using these nanocarriers. In a study reported in the literature [101], the authors developed clotrimazole-loaded solid lipid NPs, which also contained alpha-lipoic acid (able to reduce the generation of reactive oxygen species), in order to obtain a synergistic treatment for *Candida albicans* infection. Moreover, the same NPs were also coated with a cationic lipid; both uncoated and coated NPs exhibited high homogeneity, reduced mean particles size, and high physical stability with good drug entrapment into the lipid inner matrix. A slow and constant drug release occurred, without burst release; moreover, the presence of alpha-lipoic acid did not alter clotrimazole antimicrobial effectiveness, making this system a promising strategy that could also take profit of the protective effect of alpha-lipoic acid in the treatment of VC. 

Unlike solid lipid NPs, polymeric NPs do not contain a lipid matrix, but are made of synthetic or natural polymers, e.g., polyacrylamide, polyacrylate, chitosan, or gelatin, as previously reported [91]. 

In 2018, Martínez-Pérez et al. prepared clotrimazole-loaded PLGA NPs as vaginal delivery systems for antimycotic drugs, as PLGA bioadhesivity is lower than that required to achieve efficient therapy through vaginal administration. The surface of PLGA NPs was modified with chitosan to improve their mucoadhesivity. A bi-phasic drug release profile was observed for both modified and unmodified NPs for 18 days, and the presence of chitosan enhanced the microbicidal and bioadhesive properties of the carrier by increasing clotrimazole effectiveness against *Candida albicans* and the interaction with mucin. Furthermore, both modified and unmodified systems are biocompatible and safe [102]. Similar results were obtained using PCL as polymeric matrix [103].

Liposomes have been investigated for vaginal delivery of antifungal agents. As it is known, their liquid nature is unfavorable for the applicability in vaginal delivery. Therefore, mucoadhesive Carbopol resins and chitosan, which are compatible with liposomes, are frequently used to increase the viscosity of liposomal preparation for the vaginal delivery of calcein, chloramphenicol, acyclovir, clotrimazole, and metronidazole [104]. In a study by Berginc et al., curcumin-loaded chitosan/Carbopol-coated liposomes were developed to obtain the highest curcumin tissue retention and the lowest tissue permeability. Bioadhesive liposomes resulted in being a better drug delivery system for curcumin targeting vaginal mucosa than free drug solution and non-coated liposome. In fact, both chitosan and Carbopol coatings significantly increased curcumin permeability through vaginal mucus in vitro. These results make liposomes an interesting carrier over conventional dosage forms [105]. Liposomes were also included in a tablet dosage form, suitable for vaginal administration, as reported in a study by de Jesús Valle et al. (2018), wherein a liposomal formulation was designed and evaluated for the simultaneous delivery of fluconazole and itraconazole with d-alpha-tocopheryl polyethylene glycol 1000 succinate, a safe adjuvant approved by the FDA, used as an emulsifier, solubilizer, antioxidant, and vitamin E supplement. Due to its surfactant effects, this additive promotes tablet disintegration, leading to a rapid release of both fluconazole and itraconazole and facilitating the prevention of fungal biofilm [106].

Micro- and nanocapsules are further interesting drug delivery systems for antifungals, especially via transmucosal administration, since they ensure high surface area, small size, improved stability of incorporated drugs, ability to protect poorly water-soluble drugs against chemical or enzymatic degradation, enhanced bioavailability, and controlled release [107]. Chitosan-coated nanocapsules containing tioconazole and econazole were developed for vaginal administration, revealing high encapsulation efficiency (more than 87%), stability over two months, and antifungal activity against *Candida albicans* at non-toxic concentration for human keratinocyte cell line [108]. Moreover, microcapsules, offering uniform dispersion in the targeted site, reproducible drug adsorption, and reduced local irritation, were also used for the delivery of some hydroalcoholic extracts with proved antioxidant and antifungal activity. In a study by Moreno et al. [107], electrosprayed chitosan-based microparticles were developed for the vaginal administration of selected medicinal plant extracts whose biological properties were not affected by the system. A different release rate of the compounds, better solubility of the extracts, and increased bioavailability in the vaginal environment were observed. When poloxamers were present in microparticles formulation, drug solubility/availability and microparticle bioadhesion to the mucosal tissue increased. In fact, their ability to adhere to the vaginal mucosa for a prolonged period of time improved the treatment of localized infection, reducing administration frequency compared to commercial vaginal cream [109]. Gupta et al. [110] developed vaginal tablets containing clotrimazole-loaded microspheres using bioadhesive polymers such as hydroxypropyl methylcellulose, sodium carboxymethyl cellulose, and Carbopol to obtain a prolonged therapeutic activity at the site of infection. In vitro studies of bioadhesive strength and adhesion time showed the capacity of these tablets to adhere to the vaginal mucosa for a long period of time, improving drug availability. Consequently, the formulation is easy to administer, simple, and comfortable, allowing for a possible controlled clotrimazole release.

Micro- and nanocapsules might also be used to load films and, as for the above-cited treatment of viral infections, they can be considered a promising approach in treatment of VC.

Bassi et al. (2015) developed carboxymethyl derivative or fenugreek gum, a galactomannan polysaccharide obtained from seeds of *Trigonella foenum-graecum*, which possess film forming properties and bioadhesive potential. Then, films were loaded with nystatin demonstrating good hydrophilic and swelling properties. Nystatin-loaded film with 5% wt/wt carboxymethyl derivative or fenugreek gum and 2% v/v glycerol can provide 100% drug release over 5 h, demonstrating that drug release mechanism is a combination of diffusion and swelling. This system is non-toxic to vaginal mucosa with good antifungal properties in vivo, representing a compact, elegant, and patient acceptable dosage form for mucosal administration [111]. Recently, Calvo et al. (2019) developed drug-loaded vaginal films composed of chitosan alone or in association with hydroxypropyl methylcellulose using different amounts of PEG as plasticizer. These films are able to swell for 24 h without disintegration and they exhibit faster activity against *Candida albicans* when compared to free drug or drug-loaded ovules. Better results were obtained in the presence of hydroxypropyl methylcellulose as a controlled drug release, and a sustained antimicrobial activity was observed. Furthermore, this formulation did not produce cytotoxic effects, making it a promising alternative dosage form for the treatment of VC [112]. 

Despite the many advantages of films, emerging electrospun nanofibers seem to be even more promising as vaginal delivery systems, as reported in a work by Nematpour et al. (2020) [113]. In this study, clotrimazole-loaded nanofibers fabricated with an innovative mucoadhesive formulation of dextran and alginate were developed. Alginate was chosen for its biodegradability, non-immunogenicity, hydrophilic properties, and pH sensitivity. Moreover, to avoid the formation of beads or undesirable fibers, the addition of some solvent or surfactant to the alginate solution might be a solution. Results show that physicochemical, mechanical, and cytotoxic properties of clotrimazole-loaded nanofibers are similar to those exhibited by vaginal films, but drug-loaded nanofibers have better bioadhesion and higher antifungal properties. For this reason, the proposed system appears more favorable than its film for in vitro vaginal clotrimazole delivery. In another study, amphotericin B-loaded electrospun PLGA nanofibers were designed to eliminate systemic adverse effects, fungal resistance, drug interactions, and patient non-compliance. From the results, it appears that amphotericin B was released in a controlled manner over a prolonged period (8 days) as a result of entrapped drug diffusion and of the polymer erosion. The controlled release caused the eradication of vaginal fungal burden after 6 h in 60% of infected rats and did not induce systemic toxicity. Moreover, they did not induce fungal resistance and patient non-compliance, representing an alternative therapy for local treatment of vulvovaginal candidiasis [114].

Sponges are a dispersion of gas in a solid matrix able to create a solid porous structure, providing a potential system for local mucosal surface drug delivery. They can maintain their swollen hydrogel structure for a long period of time and allow prolonged residence time and effective drug absorption. Their porous nature and high surface area could provide a higher drug loading compared to the thin films. The efficiency of this system can be increased using mixed polymeric systems. In 2018, Shaker et al. formulated mucoadhesive vaginal sponges loaded with butoconazole nitrate, which were found to be highly stable for three months and significantly effective against vaginitis/vulvovaginitis compared to other types of sponges as well as free drug. These carriers might be an alternative tool to other commercially available vaginal antifungal systems because of their longer residence time, their improved patient compliance, and their ability to target the antifungal drug at the site of infection [115]. Microsponge matrix is formed by a huge number of interconnecting pores in a non-collapsible structure. After the application in the mucous membranes, microsponges remain in the small pores, where they slowly release the entrapped substances. They could be considered safe because they do not reach the systemic circulation. Moreover, thanks to their small pore diameter, bacterial cells with a size range from 0.007 to 0.2 μm cannot infiltrate into the microsponge cavities. Moreover, the controlled release of microsponges prevents the accumulation of a great drug amount in the application site, decreasing the potential skin irritation. Salah et al. developed a vaginal microsponge system for the delivery of miconazole, which displayed a desirable flowability and release rate with an in vitro antifungal activity comparable to the marketed product. In the presence of Carbopol, the retention of the dosage form in the vagina was further prolonged, increasing the contact time of the drug with the vaginal mucosa and improving the therapeutic efficiency. More studies on humans are required to investigate the efficacy, compliance, and side effects [116]. In another study, cellulosic derivative lyophilized sponges were developed for the delivery of cidofovir, revealing good mucoadhesive strength. The optimally lyophilization temperature should be 35 °C in order to avoid the formation of large ice crystals that could further damage the incorporated cidofovir. Moreover, if well formulated, this system could exhibit a dry solid form, be able to protect the entrapped drug, and be easy to handle, with a low rehydration speed in cervicovaginal mucus, a good mucoadhesive strength, and an optimal viscosity [117].

### 3.4. Vaginal Drug Delivery Systems for Parasitic Infections

#### Trichomoniasis

Currently, 5-nitroimidazole drugs are used for treatment of trichomoniasis by oral and parenteral routes. However, drug resistance emergence and intolerance to nitroimidazoles contribute to increase the interest in new strategies to fight against this disease. Lately, several efforts have been made in developing local treatments of trichomoniasis cases.

The most common local devices used in the treatment of trichomoniasis display several disadvantages for microbicide delivery because they are generally limited to delivery of low molecular weight, hydrophobic drugs, e.g., dapivirine, and their production requires heating at high temperatures (80–160 °C), which could degrade thermally sensitive active compounds. Consequently, researchers have focused their attention on novel mucoadhesive polymer formulations and matrices to improve the biodistribution of anti-*Trichomonas* compounds. Fernando et al. (2019) developed microporous PCL matrices for the vaginal delivery of tinidazole. In vitro experiments show high loading of tinidazole that is gradually released (until 50%) over 7 days into simulated vaginal fluid with highly retained antiprotozoal activity. However, additional in vitro studies are necessary to evaluate the flexural properties and the insertion/retention characteristics of these matrices [118].

Another class of nanocarriers that could be exploited for the treatment of trichomoniasis is represented by liposomes. Li et al. developed fibrauretine-loaded propylene glycol-embodying deformable liposomes. Fibrauretine is an isoquinoline alkaloid isolated from the rattan of *Fibraurea recisa* Pierre. It has a noteworthy anti-*Trichomonas vaginalis* activity but low oil/water partition coefficient, which makes it difficult to pass through the vaginal epithelium and diffuse into the vaginal mucosa where pathogens are present. The encapsulation into deformable liposomes can enhance the permeation of fibrauretine through vaginal mucosa, allowing high in situ levels and avoiding the absorption into systemic circulation. In this way, a vaginal local targeting effect is obtained, and toxic drug adverse effects are reduced. In addition, these fibrauretine-loaded deformable liposomes are non-irritant and well tolerated in vivo. All this evidence demonstrates the promising activity of these developed systems and should especially be supported in the future [119].

Table 3 summarizes the strategies discussed above that have been developed to treat vaginal infections through the topical route. The main characteristics and applications are briefly described.

## 4. Conclusions

Conventional therapies in the treatment of vaginal infections generally consist of systemic or local administration of antimicrobial agents. However, they have several limitations, including side effects, drug resistance, and infection resistance. In this context, demand for new alternatives is increasingly relevant.

Recently developed strategies include administration of antimicrobial agents through the vaginal route providing reduced drug doses, convenient administration, and improved compliance. Unfortunately, the major challenges for intravaginal drug delivery are the low residence time and the presence of a complex mucus barrier limiting drug permeation. The advent of nanotechnology significantly enhances mucoadhesive properties and vaginal retention of local dosage forms with consequent increased drug permeation. In addition, nanoscale systems have been potentially useful in developing innovative preventive and diagnostic tools that are able to manage vulvovaginal infections.

In conclusion, this review highlights the advantages of nanomaterial-based systems that can facilitate the development of new approaches and opening new opportunities in the area of vaginal infections. 

## Figures and Tables

**Table 1:** Pathogens and treatments of vaginal infections.

Pathogen	Recommended Treatments	Alternative Treatments
Bacterial infections		
*Gardnerella vaginalis*	Metronidazole 400 mg OR 2-daily × 7 days, 0.75% metronidazole vaginal gel, or 2% clindamycin vaginal cream	Use of probiotics or prebiotics Use of essential oils Local acidification
*Chlamydia trachomatis*	Azithromycin 1 g OR doses × 7 days or doxycycline 100 mg OR × 7 days	Erythromycin, ofloxacin, levofloxacin
*Neisseria gonorrhoeae*	Ceftriaxone 250 mg IM one dose or azithromicin 1 g OR one dose	Dual antibiotic treatment Broad-spectrum antibiotics. (e.g., macrolides, tetracyclines, fluoroquinolones)
*Treponema pallidum*	Benzathine penicillin G IM 3-weekly, ceftriaxone or tetracyclines	Azithromycin
Viral infections		
*Herpes simplex*	Acyclovir, valacyclovir, or famciclovir OR doses × 7–10 days	Foscarnet or cidofovir (in case of acyclovir resistance)
*Human papilloma virus*	Topical application (cream/gel) of trichloroacetic acid or podophyllin derivatives or imiquimod	Cryotherapy, electrocautery, laser, or surgical excision
*Human immunodeficiency virus type 1*	Inhibitors of reverse transcriptase and protease	Combination of several antiviral agents; use of microbicides to prevent transmission
Fungal infections (yeasts)		
*Candida albicans*	OR or topical treatments with antimicotic agents (ketoconazole, clortrimazole, flucanozole, or echinocandins)	Dual treatment amphotericin B/flucytosine Allylamines Use of probiotics
Parasitic infections		
*Trichomonas vaginalis*	Metronidazole or tinidazole 2 g OR one dose	Disulfiram and nithiamide Local protective formulations Vaccines

**Table 2:** Some examples of commercial vaginal products for local drug delivery [30,31].

Product	Use	Dosage Form	Side Effects
Metrogel-vaginal (metronidazole)	Bacterial vaginosis (BV)	Gel	Vaginal discharge, vulvovaginal irritation, cervicitis
Vagistat-l (tioconazole)	Anti-fungal, candida infection	Ointment	Shortness of breath; swelling of lips, face, or tongue
Trivagizole (clotrimazole)	Anti-fungal infection	Cream	Partners may experience minor skin irritation
Zidoval (Metronidazole)	BV	Gel	Irritation, vaginal discharge, itching, burning, numbness, abdominal pain, to avoid in patients with blood dyscrasias
Dalacin (clindamycin)	BV	Cream	Overgrowth of non-susceptible organisms (yeasts), diarrhea, pseudomembranous colitis, interactions with protection for sexually transmitted disease/contraceptive
Flagystatin (metronidazole + nystatin)	BV	Ovules	Cross-resistance, sensitizations, vaginal burning, local irritation, spot on the skin around the knees, fatigue
Canesten (clotrimazole)	Candidiasis	Cream	Allergic reactions, blisters, discomfort, pain, edema, erythema, irritation, rash, stinging, burning
Gyno-Daktarin (miconazole nitrate)	Candidiasis	Ovules	Rash, genital pruritus, burning sensation, vulvovaginal discomfort, dysmenorrhea
Neo-Penotran (metronidazole + miconazole)	Trichomoniasis	Ovules	Vaginal irritation, GI disorders, rash, nervous system disorders
Purfem (stains of lactobacilli)	Supplement of endogenous bacteria	Ovules	No undesirable effects have been reported
FloraFemme (strains of lactobacilli and bifidobacteria)	Supplement of endogenous bacteria	Ovules	No undesirable effects have been reported

**Table 3:** Main innovative strategies for the vaginal delivery of antimicrobials agents.

System		Drugs	Characteristics	Uses
**Dendrimers** [32,120]		Efavirenz	Physiologically compatible, safe, well-tolerated, innate antiviral properties (in some cases)	Prevention of viral infection, treatment of bacterial infection (*Chlamydia trachomatis*)
**Films** [121]		Probiotic bacterial microbicides, itraconazole, metronidazole, clindamycin phosphate, dapivirine, and other anti-HIV agents	Flexible, soft, tough, non-cytotoxic in vitro, prolonged release, good peel ability, moderate tensile strength, high percentage of elongation at break, stable, bioactive	Treatment of BV, VC, trichomoniasis, HIV
**Liposomes** [32,120]		Clotrimazole, metronidazole, chloramphenicol, acyclovir, ciclopiroxolamine, curcumin, siRNA	Increasing solubility of poorly soluble drugs, prevention of drug degradation, controlled release, non-toxic	Treatment of vaginitis, prevention/treatment of viral infections (HIV, HSV), *Candida albicans* infections
**Gel**	Muchoadesive gel [122]	Clotrimazole, metronidazole, *Lactobacillus acidophilus,* econazole, miconazole, acyclovir, ciprofloxacin	Suitable mechanical and release properties, good vaginal retention, enhanced permeation/penetration, able to form a physical barrier	Treatment of vaginal infections (e.g., *Chlamydia trachomatis*), prevention of HIV infection
	Polymeric gel [31,123]	Clotrimazole, metronidazole, tenofovir, reverse transcriptase inhibitors, amphotericin B, acyclovir, cidofovir	Viscous, enhanced vagina retention, pseudoplastic or thixotropic behavior that aids dosage spreading, in some cases stimuli-responsive properties, easy production, sustained release, biocompatible	Treatment of *Chlamydia trachomatis*, HIV, sexual transmitted infections, candidiasis, genital herpes, HPV
**Nanofibers** [120]		Cisplatin, polyanionic poly-lactic acid (PLLA), tenofovir	High drug accumulation, promising outcomes, good drug release, non-cytotoxic	Treatment of vaginal infections (HIV)
**Nanoemulsions** [120]		Curcumin, clotrimazole	Very stable; good dissolution rate, solubility, and bioavailability; easily applied	Treatment of *fungi* infections
**NPs**	Polymeric NPs [32,120]	Acyclovir, tenofovir, dapivarine, raltegravir, efavirenz, paclitaxel, siRNA	Sustained release, increased uptake into cervical tissue, poor toxicity, better penetration and diffusion	Viral and sexually transmitted infections (HIV, HSV), *Candida albicans* infections
	Solid lipid NPs [32]	Tenofovir, clotrimazole	Well tolerated in in vitro models	*Fungi* vaginal infections
**Tablets** [122]		Itraconazole, clotrimazole, metronidazole, *Lactobacillus acidophilus,* ketoconazole, chlorhexidine	Loaded with microspheres; precise dosing; good drug stability; avoidance of antimicrobial agents for preservation; easy handling, storage, and administration; low cost	Treatment of vaginal infections (e.g., *Candida albicans*)

## Data Availability

Data sharing not applicable.

## Data Sources

We searched on PubMed, Web of Science, and Scopus for vaginal infection topics, and retrieved relevant references from research articles and reviews. Search terms included vaginitis, bacterial vaginosis, candida, trichomonas, vaginal viral, and fungal infections. Search dates: October–December 2020.

## Abbreviations

**AIDS**	Acquired immune deficiency syndrome
**BV**	Bacterial vaginosis
**DOX**	Doxorubicin
**FRT**	Female reproductive tract
**HIV**	*Human immunodeficiency virus*
**HPV**	*Human papilloma virus*
**HSV**	*Herpes simplex virus*
**IM**	Intramuscular
**LDH**	Layered double hydroxides
**NPs**	Nanoparticles
**OR**	Oral
**O/W**	Oil in water
**PCL**	Poly-ε-caprolactone
**PEG**	Poly(ethylene glycol)
**PEO**	Poly(ethylene oxide)
**PLGA**	Poly(lactic-*co*-glycolic acid)
**PPO**	Poly(propylene oxide)
**PVA**	Poly(vinyl alcohol)
**VC**	Vulvovaginal candidiasis
**W1/O/W2**	Water in oil in water
**W/S**	Water in silicone

## References

[1] Soderberg S.F. (1986). Vaginal disorders. Vet. Clin. N. Am. Small Anim. Pract..

[2] Siddique S.A. (2003). Vaginal Anatomy and Physiology. J. Pelvic Med. Surg..

[3] Smith S.B., Ravel J. (2017). The vaginal microbiota, host defence and reproductive physiology. J. Physiol..

[4] Prabhu A., Gardella C. (2015). Common vaginal and vulvar disorders. Med. Clin. N. Am..

[5] Ferris D.G., Litaker M.S., Woodward L., Mathis D., Hendrich J. (1995). Treatment of bacterial vaginosis: A comparison of oral metronidazole, metronidazole vaginal gel, and clindamycin vaginal cream. J. Fam. Pract..

[6] Paavonen J., Mangioni C., Martin M.A., Wajszczuk C.P. (2000). Vaginal clindamycin and oral metronidazole for bacterial vaginosis: A randomized trial. Obstet. Gynecol..

[7] Alexander N.J., Baker E., Kaptein M., Karck U., Miller L., Zampaglione E. (2004). Why consider vaginal drug administration?. Fertil. Steril..

[8] Srikrishna S., Cardozo L. (2012). The vagina as a route for drug delivery: A review. Int. Urogynecol. J. Pelvic Floor Dysfunct..

[9] Gupta S., Gabrani R., Ali J., Dang S. (2011). Exploring Novel Approaches to Vaginal Drug Delivery. Recent Pat. Drug Deliv. Formul..

[10] Vermani K., Garg S. (2000). The scope and potential of vaginal drug delivery. Pharm. Sci. Technol. Today.

[11] Mei L., Chen J., Yu S., Huang Y., Xie Y., Wang H., Pan X., Wu C. (2017). Expansible thermal gelling foam aerosol for vaginal drug delivery. Drug Deliv..

[12] Bradshaw C.S., Sobel J.D. (2016). Current Treatment of Bacterial Vaginosis-Limitations and Need for Innovation. J. Infect. Dis..

[13] Menard J.P. (2011). Antibacterial treatment of bacterial vaginosis: Current and emerging therapies. Int. J. Women Health.

[14] O’Connell C.M., Ferone M.E. (2016). Chlamydia trachomatis genital infections. Microb. Cell.

[15] Taylor B.D., Haggerty C.L. (2011). Management of Chlamydia trachomatis genital tract infection: Screening and treatment challenges. Infect. Drug Resist..

[16] Piszczek J., St. Jean R., Khaliq Y. (2015). Gonorrhea: Treatment update for an increasingly resistant organism. Can. Pharm. J..

[17] Unemo M. (2015). Current and future antimicrobial treatment of gonorrhoea—The rapidly evolving Neisseria gonorrhoeae continues to challenge. BMC Infect. Dis..

[18] Hill S.A., Masters T.L., Wachter J. (2016). Gonorrhea—An evolving disease of the new millennium. Microb. Cell.

[19] Lynn W.A., Lightman S. (2004). Syphilis and HIV: A dangerous combination. Lancet Infect. Dis..

[20] Clement M.E., Okeke N.L., Hicks C.B. (2014). Treatment of syphilis: A systematic review. JAMA J. Am. Med. Assoc..

[21] Sauerbrei A. (2016). Herpes Genitalis: Diagnosis, Treatment and Prevention. Geburtshilfe Frauenheilkd..

[22] Groves M.J. (2016). Genital herpes: A review. Am. Fam. Physician.

[23] Hathaway J.K. (2012). HPV: Diagnosis, Prevention, and Treatment. Clin. Obstet. Gynecol..

[24] Cameron D.W., D’Costa L.J., Maitha G.M., Cheang M., Piot P., Simonsen J.N., Ronald A.R., Gakinya M.N., Ndinya-Achola J.O., Brunham R.C. (1989). Female to male transmission of human immunodeficiency virus type 1: Risk factors for seroconversion in men. Lancet.

[25] Simon V., Ho D.D., Karim Q.A. (2006). HIV/AIDS epidemiology, pathogenesis, prevention, and treatment. Lancet.

[26] Prokofjeva M.M., Kochetkov S.N., Prassolov V.S. (2016). Therapy of HIV Infection: Current Approaches and Prospects. Acta Nat..

[27] Mendling W. (2015). Guideline: Vulvovaginal candidosis (AWMF 015/072), S2k (excluding chronic mucocutaneous candidosis). Mycoses.

[28] Kissinger P. (2015). Epidemiology and Treatment of Trichomoniasis. Curr. Infect. Dis. Rep..

[29] Bouchemal K., Bories C., Loiseau P.M. (2017). Strategies for Prevention and Treatment of Trichomonas vaginalis Infections. Clin. Microbiol. Rev..

[30] Hussain A., Ahsan F. (2005). The vagina as a route for systemic drug delivery. J. Control. Release.

[31] Cook M.T., Brown M.B. (2018). Polymeric gels for intravaginal drug delivery. J. Control. Release.

[32] Vanić Z., Basnet N.S. (2014). Mucosal nanosystems for improved topical drug delivery: Vaginal route of administration. J. Drug Deliv. Sci. Technol..

[33] das Neves J., Nunes R., Machado A., Sarmento B. (2015). Polymer-based nanocarriers for vaginal drug delivery. Adv. Drug Deliv. Rev..

[34] Nowak J., Laffleur F., Schnürch A.B. (2015). Preactivated hyaluronic acid: A potential mucoadhesive polymer for vaginal delivery. Int. J. Pharm..

[35] Chopra S., Motwani S.K., Iqbal Z., Talegaonkar S., Ahmad F.J., Khar R.K. (2007). Optimisation of polyherbal gels for vaginal drug delivery by Box-Behnken statistical design. Eur. J. Pharm. Biopharm..

[36] Muguet V., Seiller M., Barratt G., Ozer O., Marty J.P., Grossiord J.L. (2001). Formulation of shear rate sensitive multiple emulsions. J. Control. Release.

[37] Tedajo G.M., Seiller M., Prognon P., Grossiord J.L. (2001). pH compartmented w/o/w multiple emulsion: A diffusion study. J. Control. Release.

[38] Özer Ö., Özyazici M., Tedajo M., Taner M.S., Köseoglu K. (2007). W/O/W multiple emulsions containing nitroimidazole derivates for vaginal delivery. Drug Deliv..

[39] Seoane M.C., Gago A.P., Vázquez G., Conde N., González P., Martinez A., Martínez X., García Varela L., Herranz M., Aguiar P. (2019). Vaginal residence and pharmacokinetic preclinical study of topical vaginal mucoadhesive W/S emulsions containing ciprofloxacin. Int. J. Pharm..

[40] Pisano S., Giustiniani M., Francis L., Gonzalez D., Margarit L., Sheldon I.M., Paolino D., Fresta M., Conlan R.S., Healey G.D. (2019). Liquid crystal delivery of ciprofloxacin to treat infections of the female reproductive tract. Biomed. Microdevices.

[41] Sims L.B., Frieboes H.B., Rankins J.M.S. (2018). Nanoparticle-mediated drug delivery to treat infections in the female reproductive tract: Evaluation of experimental systems and the potential for mathematical modeling. Int. J. Nanomed..

[42] Marciello M., Rossi S., Caramella C., López C.R. (2017). Freeze-dried cylinders carrying chitosan nanoparticles for vaginal peptide delivery. Carbohydr. Polym..

[43] Sims L.B., Miller H.A., Halwes M.E., Rankins J.M.S., Frieboes H.B. (2019). Modeling of nanoparticle transport through the female reproductive tract for the treatment of infectious diseases. Eur. J. Pharm. Biopharm..

[44] Hashemi H., Varshosaz J., Fazeli H., Sharafi S.M., Mirhendi H., Chadeganipour M., Yousefi H.A., Manoochehri K., Chermahini Z.A., Jafarzadeh L. (2019). Rapid differential diagnosis of vaginal infections using gold nanoparticles coated with specific antibodies. Med. Microbiol. Immunol..

[45] Maestrelli F., Jug M., Cirri M., Kosalec I., Mura P. (2018). Characterization and microbiological evaluation of chitosan-alginate microspheres for cefixime vaginal administration. Carbohydr. Polym..

[46] Abruzzo A., Bigucci F., Cerchiara T., Saladini B., Gallucci M.C., Cruciani F., Vitali B., Luppi B. (2013). Chitosan/alginate complexes for vaginal delivery of chlorhexidine digluconate. Carbohydr. Polym..

[47] Szymanska E., Czarnomysy R., Jacyna J., Basa A., Wilczewska A.Z., Markuszewski M., Winnicka K. (2019). Could spray-dried microbeads with chitosan glutamate be considered as promising vaginal microbicide carriers? The effect of process variables on the in vitro functional and physicochemical characteristics. Int. J. Pharm..

[48] Aderibigbe B.A. (2017). Metal-based nanoparticles for the treatment of infectious diseases. Molecules.

[49] Sangare L., Morisset R., Ravaoarinoro M. (1999). In-vitro anti-chlamydial activities of free and Iiposomal tetracycline and doxycycline. J. Med. Microbiol..

[50] Yang S., Traore Y., Jimenez C., Ho E.A. (2019). Autophagy nduction and PDGFR-β knockdown by siRNA-encapsulated nanoparticles reduce chlamydia trachomatis infection. Sci. Rep..

[51] Dixit S., Singh S.R., Yilma A.N., Agee R.D., Taha M., Dennis V.A. (2014). Poly (lactic acid)-poly (ethylene glycol) nanoparticles provide sustained delivery of a Chlamydia trachomatis recombinant MOMP peptide and potentiate systemic adaptive immune responses in mice. Nanomedicine.

[52] Panyam J., Hudson J.A.W., Hudson A.P. (2014). Nanoparticles for Imaging and Treating Chlamydial Infection.

[53] Alqahtani F., Aleanizy F., Tahir E.E., Alhabib H., Alsaif R., Shazly G., Alqahtani H., Alsarra I., Mahdavi J. (2020). Antibacterial activity of chitosan nanoparticles against pathogenic N. gonorrhoea. Int. J. Nanomed..

[54] Jøraholmen M.W., Basnet P., Tostrup M.J., Moueffaq S., Basnet N.S. (2019). Localized therapy of vaginal infections and inflammation: Liposomes-in-hydrogel delivery system for polyphenols. Pharmaceutics.

[55] Vanić Ž., Basnet N.S. (2013). Nanopharmaceuticals for improved topical vaginal therapy: Can they deliver?. Eur. J. Pharm. Sci..

[56] Yang H., Li D., He R., Guo Q., Wang K., Zhang X., Huang P., Cui D. (2010). A novel quantum dots-based point of care test for syphilis. Nanoscale Res. Lett..

[57] Tang Z., Liang Z., Nong Y., Wu X., Luo H., Gao K. (2017). Application of Goldmag immune probe in timely detection of syphilis based on GIS platform. Artif. Cells Nanomed. Biotechnol..

[58] Yang D., Ma J., Zhang Q., Li N., Yang J., Raju P.A., Peng M., Luo Y., Hui W., Chen C. (2013). Polyelectrolyte-coated gold magnetic nanoparticles for immunoassay development: Toward point of care diagnostics for syphilis screening. Anal. Chem..

[59] Antoine T.E., Mishra Y.K., Trigilio J., Tiwari V., Adelung R., Shukla D. (2012). Prophylactic, therapeutic and neutralizing effects of zinc oxide tetrapod structures against herpes simplex virus type-2 infection. Antivir. Res..

[60] Jaishankar D., Shukla D. (2016). Genital herpes: Insights into sexually transmitted infectious disease. Microb. Cell.

[61] Ensign L.M., Tang B.C., Wang Y.-Y., Tse T.A., Hoen T., Cone R., Hanes J. (2012). Mucus-Penetrating Nanoparticles for Vaginal Drug Delivery Protect Against Herpes Simplex Virus. Sci. Transl. Med..

[62] Orlowski P., Tomaszewska E., Gniadek M., Baska P., Nowakowska J., Sokolowska J., Nowak Z., Donten M., Celichowski G., Grobelny J. (2014). Tannic acid modified silver nanoparticles show antiviral activity in herpes simplex virus type 2 infection. PLoS ONE.

[63] Orłowski P., Kowalczyk A., Tomaszewska E., Ranoszek-Soliwoda K., Węgrzyn A., Grzesiak J., Celichowski G., Grobelny J., Eriksson K., Krzyzowska M. (2018). Antiviral activity of tannic acid modified silver nanoparticles: Potential to activate immune response in herpes genitalis. Viruses.

[64] Hu R.L., Li S.R., Kong F.J., Hou R.J., Guan X.L., Guo F. (2014). Inhibition effect of silver nanoparticles on herpes simplex virus 2. Genet. Mol. Res..

[65] Steinbach J.M., Weller C.E., Booth C.J., Saltzman W.M. (2012). Polymer nanoparticles encapsulating siRNA for treatment of HSV-2 genital infection. J. Control. Release.

[66] Udofot O., Jaruszewski K., Spencer S., Agyare E. (2014). Development of a novel approach to enhance the solubility of ftibamzone formulation. Integr. Mol. Med..

[67] Ramyadevi D., Rajan K.S., Vedhahari B.N., Ruckmani K., Subramanian N. (2016). Heterogeneous polymer composite nanoparticles loaded in situ gel for controlled release intra-vaginal therapy of genital herpes. Colloids Surf. B Biointerfaces.

[68] Jøraholmen M.W., Basnet P., Acharya G., Basnet N.S. (2017). PEGylated liposomes for topical vaginal therapy improve delivery of interferon alpha. Eur. J. Pharm. Biopharm..

[69] Zhen S., Liu Y., Lu J., Tuo X., Yang X., Chen H., Chen W., Li X. (2020). Human papillomavirus Oncogene Manipulation Using Clustered Regularly Interspersed Short Palindromic Repeats/Cas9 Delivered by pH-Sensitive Cationic Liposomes. Hum. Gene Ther..

[70] Furst T., Dakwar G.R., Zagato E., Lechanteur A., Remaut K., Evrard B., Braeckmans K., Piel G. (2016). Freeze-dried mucoadhesive polymeric system containing pegylated lipoplexes: Towards a vaginal sustained released system for siRNA. J. Control. Release.

[71] Wang J., Wang Y., Wang Z., Wang F., He J., Yang X., Xie W., Liu Y., Zhang Y. (2019). A thermosensitive gel based on w1/o/w2 multiple microemulsions for the vaginal delivery of small nucleic acid. Drug Deliv..

[72] Köllner S., Nardin I., Markt R., Griesser J., Prüfert F., Bernkop-Schnürch A. (2017). Self-emulsifying drug delivery systems: Design of a novel vaginal delivery system for curcumin. Eur. J. Pharm. Biopharm..

[73] Mesquita L., Galante J., Nunes R., Sarmento B., Neves J. (2019). Das Pharmaceutical vehicles for vaginal and rectal administration of anti-hivmicrobicide nanosystems. Pharmaceutics.

[74] Chaowanachan T., Krogstad E., Ball C., Woodrow K.A. (2013). Drug Synergy of Tenofovir and Nanoparticle-Based Antiretrovirals for HIV Prophylaxis. PLoS ONE.

[75] das Neves J., Araújo F., Andrade F., Michiels J., Ariën K.K., Vanham G., Amiji M., Bahia M.F., Sarmento B. (2013). In vitro and Ex Vivo evaluation of polymeric nanoparticles for vaginal and rectal delivery of the anti-HIV drug dapivirine. Mol. Pharm..

[76] Sáenz M.A., Espina M., Calpena A., Gómara M.J., Pomeda I.P., Haro I., García M.L. (2018). Design, Characterization, and Biopharmaceutical Behavior of Nanoparticles Loaded with an HIV-1 Fusion Inhibitor Peptide. Mol. Pharm..

[77] Kim S., Traore Y.L., Ho E.A., Shafiq M., Kim S.H., Liu S. (2018). Design and development of pH-responsive polyurethane membranes for intravaginal release of nanomedicines. Acta Biomater..

[78] Reis C.C., Machado A., Barreiros L., Araújo F., Nunes R., Seabra V., Ferreira D., Segundo M.A., Sarmento B., das Neves J. (2016). Nanoparticles-in-film for the combined vaginal delivery of anti-HIV microbicide drugs. J. Control. Release.

[79] Machado A., Reis C.C., Araújo F., Nunes R., Seabra V., Ferreira D., das Neves J., Sarmento B. (2016). Development and in vivo safety assessment of tenofovir-loaded nanoparticles-in-film as a novel vaginal microbicide delivery system. Acta Biomater..

[80] Cautela M.P., Moshe H., Sosnik A., Sarmento B., das Neves J. (2019). Composite films for vaginal delivery of tenofovir disoproxil fumarate and emtricitabine. Eur. J. Pharm. Biopharm..

[81] Illana A.M., Luna R.C., Pérez F.N., Bedoya L.M., Caro R.R., Veiga M.D. (2019). Freeze-dried bioadhesive vaginal bigels for controlled release of Tenofovir. Eur. J. Pharm. Sci..

[82] Yang T.T., Cheng Y.Z., Qin M., Wang Y.H., Yu H.L., Wang A.L., Zhang W.F. (2017). Thermosensitive Chitosan Hydrogels Containing Polymeric Microspheres for Vaginal Drug Delivery. BioMed Res. Int..

[83] Tian W., Han S., Huang X., Han M., Cao J., Liang Y., Sun Y. (2019). LDH hybrid thermosensitive hydrogel for intravaginal delivery of anti-HIV drugs. Artif. Cells Nanomed. Biotechnol..

[84] Mirani A., Kundaikar H., Velhal S., Patel V., Bandivdekar A., Degani M., Patravale V. (2019). Tetrahydrocurcumin-loaded vaginal nanomicrobicide for prophylaxis of HIV/AIDS: In silico study, formulation development, and in vitro evaluation. Drug Deliv. Transl. Res..

[85] Gupta P.N., Pattani A., Curran R.M., Kett V.L., Andrews G.P., Morrow R.J., Woolfson A.D., Malcolm R.K. (2012). Development of liposome gel based formulations for intravaginal delivery of the recombinant HIV-1 envelope protein CN54gp140. Eur. J. Pharm. Sci..

[86] Faria M.J., Machado R., Ribeiro A., Gonçalves H., Oliveira M.E.C.D.R., Viseu T., das Neves J., Lúcio M. (2019). Rational development of liposomal hydrogels: A strategy for topical vaginal antiretroviral drug delivery in the context of HIV prevention. Pharmaceutics.

[87] Mourtas S., Mao J., Parsy C.C., Storer R., Klepetsanis P., Antimisiaris S.G. (2010). Liposomal Gels for Vaginal Delivery of the Microbicide Mc-1220: Preparation and in Vivo Vaginal Toxicity and Pharmacokinetics. Nano Life.

[88] Wang L., Sassi A.B., Patton D., Isaacs C., Moncla B.J., Gupta P., Rohan L.C. (2012). Development of a liposome microbicide formulation for vaginal delivery of octylglycerol for HIV prevention. Drug Dev. Ind. Pharm..

[89] Krogstad E.A., Woodrow K.A. (2014). Manufacturing scale-up of electrospun poly (vinyl alcohol) fibers containing tenofovir for vaginal drug delivery. Int. J. Pharm..

[90] Krogstad E.A., Ramanathan R., Nhan C., Kraft J.C., Blakney A.K., Cao S., Ho R.J.Y., Woodrow K.A. (2017). Nanoparticle-releasing nanofiber composites for enhanced in vivo vaginal retention. Biomaterials.

[91] Sawant B., Khan T. (2017). Recent advances in delivery of antifungal agents for therapeutic management of candidiasis. Biomed. Pharmacother..

[92] Patel V.P., Damasiya H.M., Kapupara P., Ashara K.C. (2019). Temperature-dependent in Situ Gel of Clotrimazole: An Experimental Study. Folia Med..

[93] Ci T., Yuan L., Bao X., Hou Y., Wu H., Sun H., Cao D., Ke X. (2018). Development and anti-Candida evaluation of the vaginal delivery system of amphotericin B nanosuspension-loaded thermogel. J. Drug Target..

[94] Querobino S.M., de Faria N.C., Vigato A.A., da Silva B.G.M., Machado I.P., Costa M.S., Costa F.N., de Araujo D.R., Alberto-Silva C. (2019). Sodium alginate in oil-poloxamer organogels for intravaginal drug delivery: Influence on structural parameters, drug release mechanisms, cytotoxicity and in vitro antifungal activity. Mater. Sci. Eng. C.

[95] Deshkar S.S., Palve V.K. (2019). Formulation and development of thermosensitive cyclodextrin-based in situ gel of voriconazole for vaginal delivery. J. Drug Deliv. Sci. Technol..

[96] Ellah N.H.A., Aleem J.A.A., Abdo M.N., Ghadir O.F.A., Zahran K.M., Hetta H.F. (2019). Efficacy of ketoconazole gel-flakes in treatment of vaginal candidiasis: Formulation, in vitro and clinical evaluation. Int. J. Pharm..

[97] Talaat S.M., Elnaggar Y.S.R., Abdalla O.Y. (2019). Lecithin Microemulsion Lipogels Versus Conventional Gels for Skin Targeting of Terconazole: In Vitro, Ex Vivo, and In Vivo Investigation. AAPS PharmSciTech.

[98] Ruiz J.L.S., Capmany A.C.C., Enrich C.C., Febrer N.B.d., Carbó J.S., Souto E.B., Naveros B.C. (2019). Biopharmaceutical profile of a clotrimazole nanoemulsion: Evaluation on skin and mucosae as anticandidal agent. Int. J. Pharm..

[99] Hussain A., Singh V.K., Singh O.P., Shafaat K., Kumar S., Ahmad F.J. (2016). Formulation and optimization of nanoemulsion using antifungal lipid and surfactant for accentuated topical delivery of Amphotericin B. Drug Deliv..

[100] Suñer J., Calpena A.C., Clares B., Cañadas C., Halbaut L. (2017). Development of Clotrimazole Multiple W/O/W Emulsions as Vehicles for Drug Delivery: Effects of Additives on Emulsion Stability. AAPS PharmSciTech.

[101] Carbone C., Fuochi V., Zielińska A., Musumeci T., Souto E.B., Bonaccorso A., Puglia C., Petronio Petronio G., Furneri P.M. (2020). Dual-drugs delivery in solid lipid nanoparticles for the treatment of Candida albicans mycosis. Colloids Surf. B Biointerfaces.

[102] Pérez B.M., Guerrero D.Q., Tapia M.T., Tamayo R.C., Zaragoza M.L.Z., Alcalá S.A., Muñoz N.M., Segundo E.P. (2018). Controlled-release biodegradable nanoparticles: From preparation to vaginal applications. Eur. J. Pharm. Sci..

[103] Lucena P.A., Nascimento T.L., Gaeti M.P.N., de Ávila R.I., Mendes L.P., Vieira M.S., Fabrini D., Amaral A.C., Lima E.M. (2018). In vivo vaginal fungal load reduction after treatment with itraconazole-loaded polycaprolactone-nanoparticles. J. Biomed. Nanotechnol..

[104] Karimunnisa S., Atmaram P. (2013). Mucoadhesive nanoliposomal formulation for vaginal delivery of an antifungal. Drug Dev. Ind. Pharm..

[105] Berginc K., Suljaković S., Škalko-Basnet N., Kristl A. (2014). Mucoadhesive liposomes as new formulation for vaginal delivery of curcumin. Eur. J. Pharm. Biopharm..

[106] de Jesús Valle M.J., Coutinho P., Ribeiro M.P., Navarro A.S. (2018). Lyophilized tablets for focal delivery of fluconazole and itraconazole through vaginal mucosa, rational design and in vitro evaluation. Eur. J. Pharm. Sci..

[107] Moreno M.A., Mascaraque L.G.G., Arias M., Zampini I.C., Sayago J.E., Ramos L.L.P., Schmeda-Hirschmann G., López-Rubio A., Isla M.I. (2018). Electrosprayed chitosan microcapsules as delivery vehicles for vaginal phytoformulations. Carbohydr. Polym..

[108] Calvo N.L., Sreekumar S., Svetaz L.A., Lamas M.C., Moerschbacher B.M., Leonardi D. (2019). Design and characterization of chitosan nanoformulations for the delivery of antifungal agents. Int. J. Mol. Sci..

[109] Albertini B., Passerini N., di Sabatino M., Vitali B., Brigidi P., Rodriguez L. (2009). Polymer-lipid based mucoadhesive microspheres prepared by spray-congealing for the vaginal delivery of econazole nitrate. Eur. J. Pharm. Sci..

[110] Gupta N.V., Natasha S., Getyala A., Bhat R.S. (2013). Bioadhesive vaginal tablets containing spray dried microspheres loaded with clotrimazole for treatment of vaginal Candidiasis. Acta Pharm..

[111] Bassi P., Kaur G. (2015). Bioadhesive vaginal drug delivery of nystatin using a derivatized polymer: Development and characterization. Eur. J. Pharm. Biopharm..

[112] Calvo N.L., Svetaz L.A., Alvarez V.A., Quiroga A.D., Lamas M.C., Leonardi D. (2019). Chitosan-hydroxypropyl methylcellulose tioconazole films: A promising alternative dosage form for the treatment of vaginal candidiasis. Int. J. Pharm..

[113] Nematpour N., Moradipour P., Zangeneh M.M., Arkan E., Abdoli M., Behbood L. (2020). The application of nanomaterial science in the formulation a novel antibiotic: Assessment of the antifungal properties of mucoadhesive clotrimazole loaded nanofiber versus vaginal films. Mater. Sci. Eng. C.

[114] Souza R.O., Henrique de Lima T., Oréfice R.L., de Freitas Araújo M.G., de Lima Moura S.A., Magalhães J.T., da Silva G.R. (2018). Amphotericin B-Loaded Poly (lactic-co-glycolic acid) Nanofibers: An Alternative Therapy Scheme for Local Treatment of Vulvovaginal Candidiasis. J. Pharm. Sci..

[115] Shaker D.S., Ismail S., Hamed S., El-Shishtawy E.M. (2018). Butoconazole nitrate vaginal sponge: Drug release and antifungal efficacy. J. Drug Deliv. Sci. Technol..

[116] Salah S., Awad G.E.A., Makhlouf A.I.A. (2018). Improved vaginal retention and enhanced antifungal activity of miconazole microsponges gel: Formulation development and in vivo therapeutic efficacy in rats. Eur. J. Pharm. Sci..

[117] Furst T., Piette M., Lechanteur A., Evrard B., Piel G. (2015). Mucoadhesive cellulosic derivative sponges as drug delivery system for vaginal application. Eur. J. Pharm. Biopharm..

[118] Fernando H.V., Chan L.L., Dang N., Santhanes D., Banneheke H., Nalliah S., Coombes A.G.A. (2019). Controlled delivery of the antiprotozoal agent (tinidazole) from intravaginal polymer matrices for treatment of the sexually transmitted infection, trichomoniasis. Pharm. Dev. Technol..

[119] Li W.Z., Hao X.L., Zhao N., Han W.X., Zhai X.F., Zhao Q., Wang Y.E., Zhou Y.Q., Cheng Y.C., Yue Y.H. (2016). Propylene glycol-embodying deformable liposomes as a novel drug delivery carrier for vaginal fibrauretine delivery applications. J. Control. Release.

[120] Iqbal Z., Dilnawaz F. (2019). Nanocarriers for Vaginal Drug Delivery. Recent Pat. Drug Deliv. Formul..

[121] Machado R.M., de-Oliveira A.P., de-Oliveira J.M., de-Oliveira R.P. (2013). Vaginal films for drug delivery. J. Pharm. Sci..

[122] Caramella C.M., Rossi S., Ferrari F., Bonferoni M.C., Sandri G. (2015). Mucoadhesive and thermogelling systems for vaginal drug delivery. Adv. Drug Deliv. Rev..

[123] Veiga M.-D., Caro R.R., Illana A.M., Pérez F.N., Luna R.C., Thakur V., Thakur M. (2018). Polymer Gels in Vaginal Drug Delivery Systems. Polymer Gels. Gels Horizons: From Science to Smart Materials.

